# Selection of Appropriate Reference Genes for Gene Expression Analysis under Abiotic Stresses in *Salix viminalis*

**DOI:** 10.3390/ijms20174210

**Published:** 2019-08-28

**Authors:** Valentin Ambroise, Sylvain Legay, Gea Guerriero, Jean-Francois Hausman, Ann Cuypers, Kjell Sergeant

**Affiliations:** 1 Environmental Research and Innovation (ERIN) Department, Luxembourg Institute of Science and Technology (LIST) 5 Avenue des Hauts-Fourneaux, L-4362 Esch/Alzette, Luxembourg; 2 Centre for Environmental Sciences, Hasselt University, Agoralaan Building D, B-3590 Diepenbeek, Belgium

**Keywords:** real-time quantitative PCR, reference gene, BestKeeper, geNorm, GrayNorm, NormFinder, RankAggreg, *Salix*, abiotic stress

## Abstract

*Salix viminalis* is a fast growing willow species with potential as a plant used for biomass feedstock or for phytoremediation. However, few reference genes (RGs) for quantitative real-time polymerase chain reaction (qPCR) are available in *S. viminalis*, thereby limiting gene expression studies. Here, we investigated the expression stability of 14 candidate reference genes (RGs) across various organs exposed to five abiotic stresses (cold, heat, drought, salt, and poly-metals). Four RGs ranking algorithms, namely geNorm^PLUS^, BestKeeper, NormFinder, and GrayNorm were applied to analyze the qPCR data and the outputs were merged into consensus lists with RankAggreg, a rank aggregation algorithm. In addition, the optimal RG combinations were determined with geNorm^PLUS^ and GrayNorm. The genes that were the most stable in the roots were *TIP41* and *CDC2*. In the leaves, *TIP41* was the most stable, followed by *EF1b* and *ARI8*, depending on the condition tested. Conversely, *GAPDH* and *β-TUB*, two genes commonly used for qPCR data normalization were the least stable across all organs. Nevertheless, both geNorm^PLUS^ and GrayNorm recommended the use of a combination of genes rather than a single one. These results are valuable for research of transcriptomic responses in different *S. viminalis* organs.

## 1. Introduction

Perennial woody plants are present across the world and cover about 30% of the land surface [1]. Trees are a source of numerous economical products, such as timber, fuel, food, resins, and oil, but they also provide a range of ecosystem services including carbon storage, maintenance of wildlife habitats, and soil stabilization [2]. Unlike herbaceous plants, they usually have a longer life cycle, spanning from decades to centuries. Therefore, they have to withstand numerous challenges throughout their life span, under the form of both biotic and abiotic stress constraints. Abiotic stresses are the main factors affecting plant distribution and primary production [3]. From this perspective, unravelling stress acclimation and resistance mechanisms could help us to optimize plant primary production [4].

Basket willow (*Salix viminalis* L.) is a fast-growing tree species with a wide Eurasian distribution that can be found in cool, temperate, and boreal climates. It is a pioneer species of riparian environments with a moderate to high metal tolerance [5,6]. It is one of the most important species used in basketry and its fast growth makes it amongst the most used species in intensive short-rotation agro-forestry systems in northern temperate regions [7]. In addition to this, its highly developed root system and important evapotranspiration rate make *S. viminalis* highly suitable for phytoremediation, on-site metal phytoextraction and the treatment of wastewater, sewage sludge and landfill leachate [8,9]. However, while *S. viminalis* is well studied at a physiological level, it is poorly characterized at a molecular level, making breeding programs and clone selection time consuming. For example, field trials to assess *Salix* performance in soils contaminated with metals can take up to five years [10]. In addition, most of the studies focus on the aboveground organs, which represent the most valued parts but not the primary site of exposure to metals. Roots, which are directly exposed to soil and hence high concentrations of metals, act as a metal filter and in most species the bulk of metal ions are sequestered in the roots, limiting their progress into the aboveground organs via the xylem [11]. In addition, genes influencing the root system architecture are poorly known. Nevertheless, the root system architecture plays a primordial role in plant fitness as it determines the capacity of a plant to access mineral ions and water [12,13]. Information about the expression patterns of genes involved in root architecture and stress tolerance at the root level would hasten clone selection and pave the way for the development of trees with improved capacity of metal sequestration [14].

Gene expression analysis has been applied to unravel biological mechanisms in many organisms. Amongst the techniques available to analyze gene expression, real-time or quantitative polymerase chain reaction (qPCR) has been an inestimable source of data, thanks to its accuracy, specificity, high sensitivity, repeatability, and reproducibility. As such, Minimum Information for the publication of real-time Quantitative PCR Experiments (MIQE) was written in an effort to standardize qPCR practice and reduce the publication of incorrect qPCR data [15]. These guidelines include considerations on nomenclature, concepts, sample preparation, and normalization. The normalization step involves the comparison of mRNA ratios of genes of interest (GOIs) to those of reference genes (RGs). Reference genes are genes with a stable expression level across the studied conditions and are typically genes involved in basic cellular processes [16]. For proper gene expression normalization, more than one RG should be used and several algorithms exist to select the most suitable ones [17]. For the moment, no “universal reference gene” is known, necessitating the description and validation of RGs depending on the species, organs, and conditions to which plants are exposed in a study. However, stability validation of RGs is not systematic in plant science, resulting in poor data normalization, which can lead to inadequate conclusions [18,19,20].

To our knowledge, no systematic study aiming to validate potential RGs has been conducted for *S. viminalis*. Given the potential of *S. viminalis* in phytoremediation and energy production, it is essential to identify stably expressed RGs in different organs and across several conditions to foster its selection in future applications. In this work, we studied the expression pattern of 14 candidate reference genes in the roots and leaves of *S. viminalis* exposed to different conditions. In addition, we investigated the expression stability of these candidate reference genes in roots stele, roots cortex, stem xylem, and bark under control conditions. Using different algorithms and combinations thereof, we subsequently determined the most stable reference genes, produced a consensus list and the optimal combination of RGs, with an emphasis on those that could be used for studies at the root level.

## 2. Results

### 2.1. Culture Condition

Plants were exposed to salt, cold, heat, and drought stress for up to 12 days or to a mixture of metals for two months, and fine roots and leaves were sampled at different time points for RNA extraction (Table 1). In addition to fine roots and leaves, the stele and cortex of coarse roots, stem cortex and bark were collected from control plants on the 12th day to study the stability of the candidate reference genes in various organs under control condition. These samples were not taken into account when computing the stability of the candidate RGs in the roots and or the leaves.

Relative water content of control- and drought-treated soil were measure at 0,1,4,6, and 7 days after stress exposure for each plant sampled. Chlorophyll fluorescence of young leaves was measured at each sampling time, and the F_V_/F_M_ ratio was calculated as an easily obtainable stress marker [21]. Two different trends were observed in the chlorophyll fluorescent values (Table 1). Plants exposed to cold stress showed an initial decrease in F_V_/F_M_, followed by a subsequent recovery. On the contrary, the F_V_/F_M_ ratio of plants exposed to drought and heat stress decreased from the start of the experiment throughout the whole duration of the experiment. Plants rooted in metal contaminated soil for two months had the lowest F_V_/F_M_ ratio and their leaves showed signs of mild chlorosis. The precise chlorophyll fluorescence values for all plants can be found in Table S1.

### 2.2. Strategy for Data Analysis

A total of 14 candidate RGs were tested, including nine genes traditionally used as RG [*α-TUB* (α-tubulin), *β-TUB* (β-tubulin), *ACT* (actin), *ARI8* (ubiquitin protein ligase E3), *CDC2* (cyclin dependent kinase), *CYP* (cyclophilin), *EF1b* (elongation factor 1β), *eTIF5* (eukaryotic translation initiation factor 5A), GAPDH (glyceraldehyde 3-phosphate dehydrogenase), *TIP41* (type 2A phosphate activator), *UCEE2* (ubiquitin conjugating enzyme E2)]; and three genes previously reported as stable in closely related species: *OTUp* (OTU-like cysteine protease; Li et al. [22]), *PT1* (unknown protein expressed in the pollen tube; Pettengill et al. [23]), *VHAC* (vacuolar H^+^-ATPase subunit C; Li et al. [22])] (Table 2).

**Figure 1:**
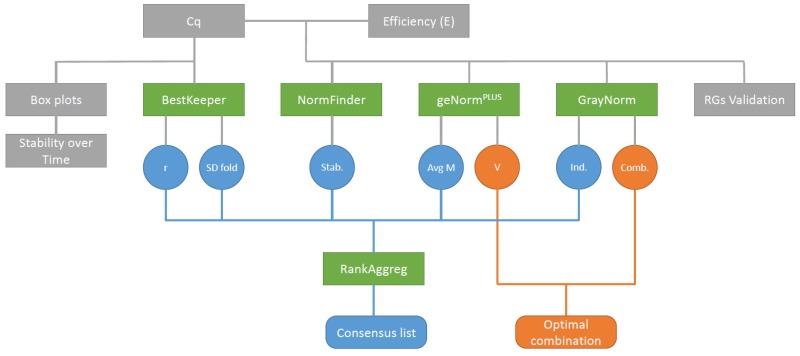
Data analysis workflow. Cycles of quantification (C_q_) and PCR efficiency (E) were determined by qPCR and then used as input for the different gene ranking algorithms. Contrary to the other ranking algorithms, BestKeeper only used C_q_ as input data. The ranking list produced by the different algorithms were then used as input data with RankAggreg to produce a consensus list. Grey boxes: raw data and descriptive statistics, green boxes: algorithms, circles: ranking algorithms output, round-cornered rectangles: final outputs, blue circles: Data used to rank gene stability, orange circles: Data used to determine the optimal gene combination. Stab.: NormFinder stability value, Avg M: Average expression stability M, Ind.: GrayNorm ranking taking only single gene combinations into account, Comb.: GrayNorm ranking taking all gene combinations into account.

The efficiency of the primers (E) was calculated (Table 2) using serial five-fold dilutions (cf. Materials and Methods, melting curves can be found in Figure S1). The quantification cycle (C_q_) was measured and descriptive statistics were performed on the raw data (Figure 1). Quantification cycles were then transformed when needed. The expression stability of the candidate RGs was assessed with four algorithms: geNorm^PLUS^ [24], BestKeeper [25], NormFinder [26], and GrayNorm [27]. We obtained a total of five lists ranking the genes by their stability and two optimal gene combinations (from geNorm^PLUS^ and GrayNorm). Since the different algorithms produced different ranking lists, we used RankAggreg to compute a consensus RGs list for each condition. In addition, we compared the optimal combinations of genes computed by geNorm^PLUS^ and GrayNorm. Validation of the RGs was performed using both the quantification cycle values and the primers efficiency.

**Figure 2:**
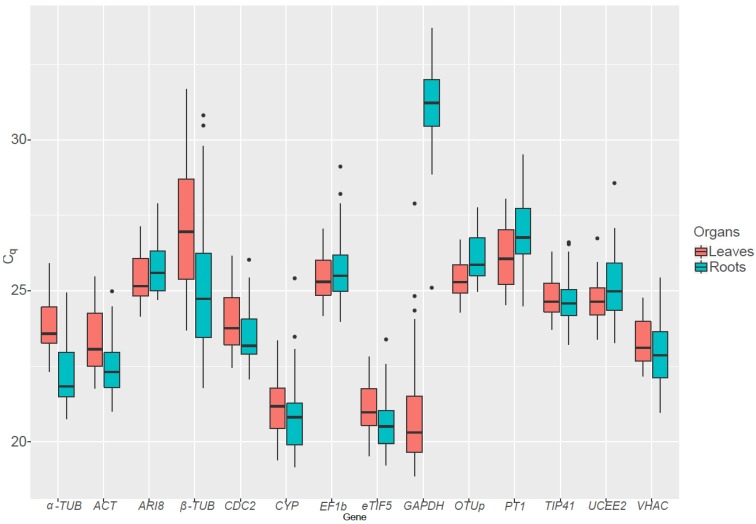
Boxplot of the quantification cycle (C_q_) values for 14 candidate reference genes in *S. viminalis* roots and leaves exposed to various abiotic stresses. Eight-month old cuttings were exposed to drought, cold, heat, metal or salt stress in triplicate for up to two months before being sampled.

Out of the 14 candidate RGs tested, *GAPDH* and *β-TUB* displayed a huge variation in their expression pattern (Figure 2). *GAPDH* is more expressed in leaves than in roots, as it has a role in the Calvin–Benson cycle in addition to is function in glycolysis. Conversely *β-TUB* is on average more expressed in the roots than in the leaves. As the presence of genes showing an important variation in C_q_ can lead to incorrect rankings [25], *GAPDH* and *β-TUB* were not taken into account when analyzing stability of RGs in all organs grouped together.

The expression level of all the candidate RGs shows a mild but non-significant increasing trend over time under control condition in both the leaves and the roots (Figures S2 and S3). While a similar trend was observed under cold and heat conditions in the roots for most candidate RGs, *CYP* and *β-TUB* show a significant increasing trend and *GAPDH* displays a decreasing trend. Trends are not that marked in the leaves exposed to cold and heat. While all candidate RGs except *CYP* show an increasing trend in the leaves of plants exposed to salt, both non-significant increasing and decreasing trends were observed in the roots of the plants exposed to the same conditions. All the genes show an increasing trend in both the leaves and the roots of plants exposed to drought condition, except for *CYP* and *VHAC* in the leaves and *GAPDH* in the roots. On the other hand, *β-TUB* displays a strong and significant increase in C_q_ values over time in leaves and roots of plants exposed to drought stress.

Expression stability of the candidate RGs was analyzed in the roots, the focus of our study. Nevertheless, we also provide RGs to use in studies focused on the leaves and/or various organ combinations in the Tables S2–S4.

### 2.3. Reference Genes Ranking

**geNorm^PLUS^** is a quantity-based algorithm with a pairwise comparison approach. Quantity-based algorithms take both C_q_ values and E into account during the computation. The key principle behind a pairwise comparison approach is that ideal RGs display similar expression patterns across the different samples. Based on this hypothesis, expression levels of ideal RGs are highly correlated between them and ideal RGs can therefore be easily detected. One drawback of this assumption is that co-regulated genes are artificially ranked amongst the most stable genes, independently of their intrinsic gene expression stability [26].

To rank RGs, geNorm^PLUS^ computes for each gene a measure of stability (named M) based on the average pairwise variation between this gene and the remaining candidate RGs. The algorithm also calculates another stability value (called Average expression stability M) by doing a stepwise exclusion of the least stable gene and averaging the M value of the remaining RGs.

In addition to ranking genes, geNorm^PLUS^ is able to determine the minimal number of RGs that should be used to obtain an accurate normalization. To do so, the software estimates the pairwise variation between two normalization factors (NF) composed of an increasing number of reference genes (namely V*_n/n+1_*, with *n* being the *n* most stable genes). A large variation indicates that the added gene (*+1*) has a significant effect on normalization and should be kept for the calculation of the NF. On the contrary, if the variation falls below a threshold value (fixed to 0.15), *n* is considered the minimum number of RGs to use for accurate normalization [24]. Alternatively, *n+1* genes can be used for normalization when V*_n/n+1_* reaches a minimum [28]. This has the advantage of providing a minimum of three RGs for normalization, which reduces the risk of having co-regulated genes.

In this experiment, *CDC2* ranked amongst the most stable genes across all the tested conditions (Table 3). TIP41 and *ACT* also ranked well, although their ranking was more variable depending on the conditions tested. *GAPDH* and *β-TUB* were among the least stable genes for each condition. *CYP* showed a variable stability pattern i.e., while it was the most stable gene under control and cold conditions, it varied between the third and twelfth position under the other conditions. The gene *eTIF5* had a similar pattern although it was generally slightly less stable than *CYP*.

**BestKeeper** is another software based on pairwise comparison. To rank the candidate RGs, the algorithm computes several stability measures, which allow the users to select the most stable genes. First, it computes the standard deviation (SD) of the C_q_ values for each gene (the lower the more stable). In parallel, it combines all the candidate RGs into an index computed based on the geometrical average of the C_q_s of all the RGs for each sample. It then estimates the coefficient of determination (r^2^) between each candidate RGs and the index (the closer to 1 the better). In addition, BestKeeper measures sample integrity by comparing for each RG, the sample C_q_ values with the index C_q_ value.

As the original excel-based algorithm allows to test a maximum of 10 genes, we used ctrlGene, a package based on R [29], after having verified it produced the same results as the original spreadsheets. When less than 10 reference genes were used and efficiency fixed to 2, the results obtained with the R-based BestKeeper package were the same as for the excel-based BestKeeper, with the exception of SD [x-fold] (data not shown). When using the efficiencies calculated from our primers, only the “[x-fold]” measures changed. This means that BestKeeper could not be classified as a quantity-based algorithm. Both the correlations and the descriptive statistics were calculated based on the C_q_ values. The efficiency factor, a quantitative factor, was then only taken into account for the calculation of the [x-fold] values [17]. As the correct [x-fold] values can easily be manually computed, we used the R package that allowed us to analyze the stability of 14 reference genes at the same time.

Based on the calculated standard deviation, *TIP41* was globally the most stable gene, followed by *CDC2* (Table 4). In six out of seven conditions tested, *GAPDH* had a SD > 1, indicating a low stability. Similarly, *β-TUB* had a SD > 1 in four conditions. Under the heat condition, *β-TUB*, *CYP* and *UCEE2* were shown to be unstable. The coefficient of determination shows that *CDC2* and *TIP41* were highly correlated to the BestKeeper index while *β-TUB* and *GAPDH* were the least correlated to it.

**NormFinder** is a quantity-model-based software. Model-based algorithms use complex statistical models to compute the variation between the expression of genes in samples belonging to different biological groups (conditions, time points, organs…). These approaches have two advantages over pairwise comparison algorithms: (1) They are theoretically less sensitive to co-regulated genes and (2) they take biological groups into account and thereby reduce the errors introduced by systematic intergroup variation [26]. However, compared to the pairwise approach, the robustness of a model-based approach is more dependent on the number of reference genes and conditions tested. For example, the NormFinder manual recommends to use a minimum of three candidate genes and two samples per group, and optimally 5–10 genes and eight samples per group.

To rank RGs, NormFinder evaluates the overall variation of the candidate RGs, but also the variation existing between sample groups. The algorithm then merges the intra- and intergroup variations values into a stability index allowing the user to select the most stable genes. The software can also give the best combination of two genes that together are the most stable.

Based on the stability values computed by NormFinder, *CDC2* was the most stable gene across all conditions (Table 5). *TIP41* was also quite stable, although its ranking varied between the second and fifth place, depending on the condition tested. Interestingly, *CYP* ranked first in two conditions (cold and metal) but eleventh under heat stress. *GAPDH* and *β-TUB* were the least stable genes across all conditions.

**GrayNorm** is another quantity-model-based algorithm specifically designed to identify RGs that limit uncertainty introduced during data normalization. GrayNorm is based on the principle that non-normalized data of GOIs contain both biologically meaningful expression differences (signal) and technical variation (noise). Signal and noise are fixed once the experiment is finished and only the NF can further change the uncertainty level. Therefore, GrayNorm identifies candidate RGs that introduce the least uncertainty during normalization.

GrayNorm works by computing the NF for each condition and each possible RG combination. It then calculates the average NF per condition in relation to the control. Finally, it orders the different RG combinations based either on the coefficient of variation of these NF averaged per condition (CV_inter_), or on the cumulative deviation of the NF [27,30]. In our study, CV_inter_ was used as the ranking factor. Interestingly, by computing all the possible RG combinations, GrayNorm can group RGs showing opposite expression variability trends, thereby producing a NF that smoothens individual gene expression variability [26].

Although *ACT* and *OTUp* rank best in most of the conditions, their stability depends highly on the conditions to which the plants are exposed. In general, *TIP41* was always found amongst the higher ranked (Table 6). The gene *ARI8* was also generally detected as stable although its rank varied importantly depending on the conditions the plants were exposed to. Interestingly, *GAPDH* was amongst the most stable genes when all the conditions were grouped together and under heat stress, where it was second and third respectively. On the other hand, *CDC2* ranked on average at the seventh place while it was determined as one of the most stable genes with the other algorithms.

### 2.4. Consensus Ranking List

In this study, four different algorithms were used to analyze the expression stability of 14 candidate RGs. However, the algorithms yielded different ranking lists due to their different approach. In order to provide a comprehensive result, we used RankAggreg [31] to compute a consensus RG list for each condition.

RankAggreg is an algorithm specifically designed to aggregate large ranking lists. To do so, it first generates all the candidate consensus lists possible. It then computes the distance between the input lists and the candidate consensus list for each of the possible candidate consensus list. Two functions can be used to compute the distance: Spearman footrule distance (which was used) or Kendall’s tau distance. Finally, RankAggreg selects the consensus list which yields the minimum distance value. As this task can be time consuming with long (>10) input lists, RankAggreg has built-in Cross-Entropy Monte Carlo and Genetic algorithms to reduce the time of computation required.

Predictably, *CDC2* and *TIP41* were amongst the most stable genes in all analyses (Table 7). *CYP* showed a high variation pattern as it ranked first in control and cold conditions but eighth and twelfth when all conditions were grouped and under heat stress, respectively. *GAPDH* and *β-TUB* were the least stable genes across all conditions, which is expected as they were ranked last by most algorithms.

### 2.5. Optimal Combination of Reference Genes to Use

Out of the four algorithms used, two (geNorm^PLUS^ and GrayNorm) allow to compute the optimum combination of reference genes to use (cf. *supra* for the principles).

**Figure 3:**
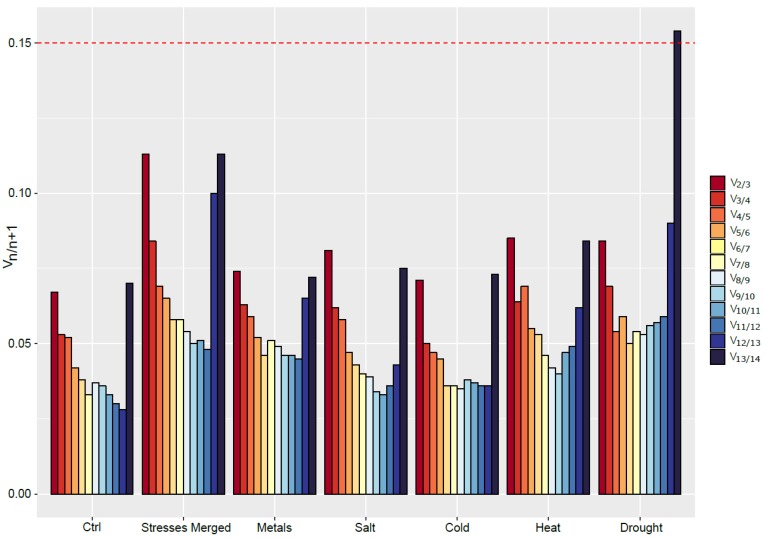
Determination of the appropriate number of RGs to use for data normalization in *S. viminalis* roots under various abiotic stresses conditions (metal, salt, cold, heat, drought) as computed by geNorm^PLUS^. The recommended threshold of 0.15 (red dashed line) was kept in this study.

Based on our data, geNorm^PLUS^ recommends the use of two reference genes to normalize the expression of GOI (Figure 3). Indeed, for every condition, the value of V_2_/V_3_ is under the threshold value of 0.15. However, based on the recommendations of Ling and Salvaterra [28], more genes should be used. For example, under control condition, the eight most stable genes could be used to normalize gene expression. However, the optimal number of RGs to use for normalization is highly dependent on the conditions studied, and ranges from four under heat stress to 11 for salt-exposed roots.

Based on our data, GrayNorm recommends to use between one and five reference genes to normalize GOIs expression values (Table 8). The number of reference genes to use for optimal normalization was highly dependent on the condition, it was one, for roots of plants exposed to metals and under control condition, two, under heat stress and when grouping all the stresses together, three, under drought and salt stresses and five, under cold stress. Interestingly, RG combinations given by GrayNorm are not always the combination of the most stable genes. Most of the time, optimal combinations consist of genes classified as highly stable and genes individually classified as having lower stability values. For example, under cold conditions, GrayNorm suggests to use the four most stable genes (*OTUp*, *TIP41*, *ARI8*, *ACT*) and one less stable gene (*GAPDH*, ranked 11 out of 14).

### 2.6. Gene Stability in the Leaves and Other Organs

In the leaves, *TIP41* was the most stable gene across all conditions, according to the consensus list (cf. Table S2). *ARI8* displayed a high expression stability under most conditions but falls at the fifth and seventh place under cold and drought conditions, respectively. Similarly, *EF1b* ranked well except under heat stress and when all conditions were grouped (where it ranked at the sixth position). In the leaves, *CDC2* showed an important variation in its stability as it ranked first under salt and heat condition, but ninth under drought condition. Interestingly, *CYP* was the second least stable gene in average, while it ranked as the most stable gene in roots exposed to control and cold conditions. As in roots, *β-TUB*, *GAPDH* and *PT1* were amongst the least stable genes.

When the data from the roots and the leaves were merged together, *TIP41* was the most stably expressed gene, according to the consensus list (cf. Table S3). Under most conditions, *EF1b* and *CDC2* were stably expressed when both organs were grouped together. Apart from *β-TUB* and *GAPDH*, which displayed a huge variation in their expression pattern, *α-TUB*, *OTUp*, and *PT1* were not stable under any tested conditions. To normalize qPCR data across various organs under control conditions, *eTIF5*, *TIP41*, *CDC2*, and *EF1b* were the most stable genes (cf. Table S4). Overall, *TIP41* was the most stably expressed gene across all conditions and organs.

### 2.7. Reference Genes Validation

The validity of the candidate RGs identified was tested in the different organs and conditions by studying the expression profiles of three reporter genes: *HSP17* (a 17.6 kDa heat shock protein, SapurV1A.0393s0170), *ADC* (arginine decarboxylase, SapurV1A.0091s0150), and *CAT* (catalase, SapurV1A.0016s0660).

The small heat shock protein HSP17 has a chaperone-like activity and its expression changes when exposed to temperature and osmotic stresses [32]. Arginine decarboxylase is an enzyme playing a role in the synthesis of putrescine, a polyamine, which plays a role in the tolerance to various abiotic stresses among which osmotic stress, salinity, hypoxia, and cold [33]. The last tested gene codes for catalase, a H_2_O_2_-scavenging enzyme with expression changes in response to osmotic, temperature, and oxidative stresses [34].

It was assumed that the stress treatment would affect the expression level of the reporter genes but not the expression of the RGs. The data was analyzed with qBase^PLUS^ and normalized using *CDC2*, *TIP41*, *ARI8*, *ACT*, and *EF1b* for both the roots and the leaves (figure for the leaves are found in Figure S4), since these genes were the most stable according to the consensus list.

**Figure 4:**
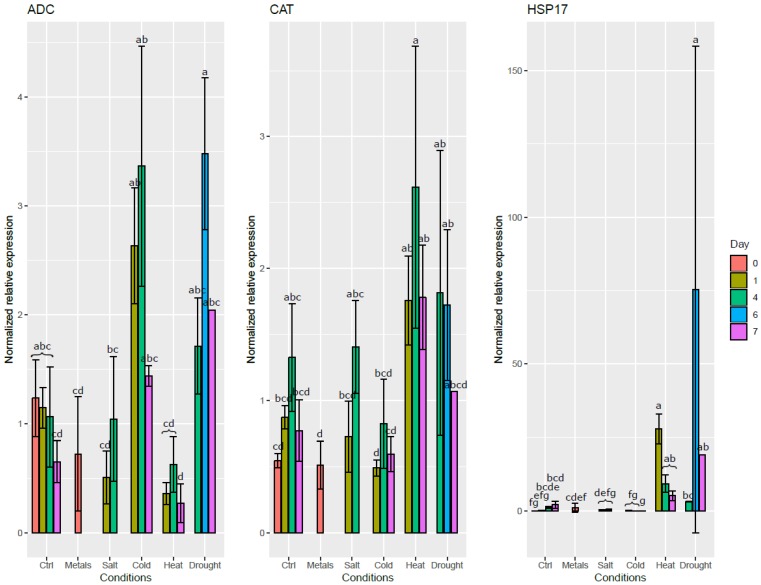
Normalized relative expression level of three stress-responsive genes in the roots of *S. viminalis* exposed to various abiotic stresses for up to two months. Data was normalized using *ACT*, *ARI8*, *CDC2*, *EF1b* and *TIP41*. Means that share a letter are not significantly different at α = 0.05.

As can be seen in Figure 4, the *ADC* expression level increased in roots exposed to cold and drought stresses. On the contrary, it decreased in the roots exposed to heat et metals. *CAT* expression was induced in the roots by both drought and heat stresses. Drought and heat stresses both significantly induced *HSP17* expression. Salt stress did not significantly affect the expression level of any of these genes in the roots, except a mild decrease of expression of *ADC* during the first day. Interestingly, salt stress had a significant effect on the expression level of both *ADC* and *HSP17* in the leaves on the first day after the stress application (Figure S4).

## 3. Discussion

Currently, qPCR is widely used to perform gene expression analysis [15]. However, the use of non-stable RGs for data normalization can lead to improper analysis and conclusions [20]. Therefore, the aim of this study was to identify RGs in *S. viminalis* with a stable expression level across various conditions and organs. While validation of appropriate RGs has been done in many plants, most studies focus on herbaceous plants [30] and no RGs are publicly available for the closely related *S. purpurea*, which has been sequenced.

Due to the high potential of *S. viminalis* in phytoremediation, together with its high primary productivity, we performed RG validation for leaves and roots exposed to various conditions. The study showed that the potential RGs had different expression profiles depending on the organ studied, which highlights the need to perform condition- and organ-specific RG validation before every gene expression experiment.

Chlorophyll fluorescence was measured and the F_V_/F_M_ ratio was calculated and used as a stress marker. Interestingly, the F_V_/F_M_ ratio of the drought-exposed plants only slightly decreased during the six first days but on the seventh, two of the three plants had lost their leaves. Conversely, the plants exposed to cold showed a drop in F_V_/F_M_ value the first day and subsequently recovered, although the stress was maintained at the same level. According to Lichtenthaler (1998) [35], stress has a dose-effect relationship i.e., while some stressors have a low effect on plant survival at low “concentration”, higher “concentration” or longer exposure time could lead to significant damage to the plant integrity (exhaustion phase). Conversely, some stresses can induce a temporary distress (alarm phase) which act as a stimulatory signal for the plant and induces its hardening. The F_V_/F_M_ ratio could indicate the alarm and exhaustion phases in the stress response rather than the application of a stressor to the plant.

Four different algorithms, geNorm^PLUS^, BestKeeper, NormFinder, and GrayNorm were used to analyze the expression stability of 14 candidate RGs. While the four algorithms produced globally similar rankings, some punctual but important differences could be observed between the algorithms’ outputs. For example, while geNorm^PLUS^, BestKeeper and NormFinder ranked *GAPDH* amongst the least stable genes across all conditions, GrayNorm ranked this gene among the most stable ones when all conditions were grouped and under heat stress. These kinds of discrepancies arise from the fact that each algorithm is based on specific premises and working hypotheses. However, under most conditions, the same general pattern could be observed across the different algorithms. More specifically, *CDC2* and *TIP41* always ranked amongst the most stable genes across all conditions; *CYP* displayed a high expression stability under control and cold conditions but a lower expression stability under the other conditions; *ACT*, *ARI8*, and *EF1b* were globally stable; and *GAPDH*, *β-TUB*, and *PT1* were the least stable genes. Nevertheless, the variations existing between the outputs of the different algorithms were smoothened by the use of RankAggreg, which allowed us to produce a consensus list.

In the consensus list, *CDC2* and *TIP41* were the most stable RGs across all conditions. *CDC2* codes for a cyclin dependent kinase (CDK1/CDKA), which is involved in eukaryotic cell cycle regulation. In association with various cyclins, CDK1/CDKA is known for its role in the control of the transition from one cell cycle phase to another [36]. It is interesting that its expression remained stable during metal stress, as cadmium has been reported to block the cell cycle at the G2 checkpoint in the roots of *Lactuca sativa* [37]. *TIP41* codes for a 41kDa protein interacting with TAP42, which is involved in the regulation of cellular growth in response to the nutrient status and the environment [38]. It has been shown to be induced by long-term exposure to NaCl [38], but this was not observed in our study. *CYP* codes for a cyclophilin peptidyl prolyl isomerase involved in a wide range of basal functions, such as proper protein folding and regulation of plant growth and development. Some cyclophilin isoforms have been observed to be induced by abiotic stresses [39], but this was not the case for the cyclophilin isoform used in the present study. *UCEE2* and *ARI8* both code for proteins involved in the ubiquitination process (respectively in ubiquitin conjugation and ubiquitin ligation) [40]. Proteins involved in ubiquitination are constitutively expressed and have been reported to be reliable RGs in various species and under diverse stresses [16]. Interestingly, while *ARI8* was relatively stable, *UCEE2* expression stability varied from relatively stable (sixth most stable gene in all conditions grouped) to unstable (thirteenth under control condition). *GAPDH*, which was amongst the least stable genes, is involved in glycolysis but also in non-metabolic functions during abiotic stress [41]. It was less expressed in the roots than in the leaves, as it also plays a role in the Calvin-Benson cycle.

The expression level of the different candidate RGs was globally stable over time in the roots of *S. viminalis* under control condition. The same pattern could be observed for every RGs except *GAPDH* in the roots of plants exposed to cold and heat. No clear trend could be observed in the roots exposed to salt stress as some genes showed increasing expression level and other decreasing. Under drought conditions, a significant increase in the C_q_s value could be observed for most genes, except for *GAPDH*, which displays opposite trend. Curiously, candidate RGs that showed different trends than the other RGs (for example *CYP*, *β-TUB* and *GAPDH* in the roots under heat stress) were ranked as less stable by the algorithms. This is due to the fact that ideal RGs expression patterns are expected to be highly correlated between them.

Interestingly, *GAPDH* and *β-TUB*, which are both widely used as reference genes in plants, were ranked as the least stable genes under most conditions. This is worrisome, as standardization with non-stable RGs can lead to misinterpretations of data, all the more since often only one RG is used. For example, let us consider an experiment in which we want to investigate the effect of metal exposure on the expression level of some GOI in the roots of *S. viminalis*. If a GOI is standardized with *GAPDH*, which has a maximum variation of 4.04 C_q_ between control roots and roots exposed to metals, there could potentially be a [(amplification factor)^maximum variation^ = 1.88^4.04^ =] 12.81-fold difference in GOI quantification compared to the situation where we use an ideal RG, which would have no variation across samples and conditions. However, as mentioned previously, perfect RGs do not exist, and even *TIP41*, which was ranked as the most stable gene under metal exposure, displayed a maximum variation of 2.24-fold. As such, the use of multiple RGs is recommended, as it cancels out the variation in expression observed for individual RG [17,26].

The optimum combination of genes given by geNorm^PLUS^ and GrayNorm were not the same. This was expected, as the two algorithms have a different approach to determine the best combination of RGs. Indeed, geNorm^PLUS^ combines genes based on their individual stability i.e., it groups together the *n* most stable genes, as such the addition of a new RG (*n+1*) does not significantly affect the normalization factor. On the contrary, GrayNorm groups genes based on the stability of their NF. This can lead to the proposed use of genes with poor individual stability but which, grouped together, have a higher stability. In other words, geNorm^PLUS^ computes the combination of the best RGs while GrayNorm calculates the best combination of RGs. Nevertheless, it is clear from both the geNorm^PLUS^ and GrayNorm output that the optimal number of genes to use for gene expression normalization depends on the condition and the organ that is targeted, as it was already observed in *Arabidopsis thaliana* [42], *Petunia* hybrid [43], *Populus euphratica* [30], and *Salix psammophila* [22] amongst others. Nevertheless, a minimum of three stable genes should be sufficient for data normalization in most studies [28].

For the validation phase, we used three genes known to change in expression during exposure to abiotic stresses. In general, the stress-responsive genes displayed the same response patterns in both roots and leaves. However, some differences were observed between both organs. For example, while metals did not induce *ADC* in the roots, its expression level increased in the leaves. Similarly, while cold did not significantly altered *CAT* expression in the roots, it was significantly down-regulated in the cold-exposed leaves.

## 4. Materials and Methods

### 4.1. Plant Cultivation and Sampling

Green cuttings of approximately 15 cm and 5 mm diameter of one individual of *S. viminalis* were rooted in containers filled with a mix of 25 kg of potting soil and 17.5 kg sand under greenhouse conditions for 5 months. Shortly after bud break, rooted cuttings were transferred to pots (2 cuttings per pots) containing the same mixture as previously used. Three of these pots were supplemented with a mixture of metals under the form of metal chloride (final concentration of 3 ppm Cd, 60 ppm Ni, 1000 ppm Zn, 350 ppm Cu), and the rooted cuttings were allowed to grow for two months, forming the metal-exposed samples. After two months of growth in the greenhouse, the cuttings were subjected to abiotic stresses and sampled at several time points (cf. Table 1). Four cuttings were exposed to cold (9/7 °C, 16/8 photoperiod) and four to heat (35/25 °C, 16/8 photoperiod) and sampled after 1, 4, and 7 days. For salt stress, 200 mM of NaCl were solubilized in water and gradually added during the first day of the treatment; cuttings were subsequently watered with tap water and sampled after 1, 4, and 12 days. For drought stress, plants were left without watering and sampled at days 4, 6, and 7. Organs from plants under control conditions (no treatment) were sampled at each of these time points. For each of these treatments, organs from the two cuttings growing in the same pot were pooled as one biological replicate. At each sampling points, chlorophyll fluorescence was measured on the third fully expanded leaf using a Plant Efficiency Analyzer (Hansatech Instruments, Pentney, England). More precisely, intact leaves were dark-adapted for 30 min and then minimal fluorescence yield (F_0_) was determined with a diode emitting red light (650 nm). Maximal fluorescence yield (F_M_) was determined after exposure to a saturating flash of light. The variable fluorescent yield (F_V_) was calculated as F_M_ – F_0_, and the F_V_/F_M_ ratio was used as a stress marker. In addition, the volumetric water content (RWC) was recorded for the pots of control and drought-exposed plants at each sampling time with a Field Scout Digital Moisture Sensor (Turf-Tec International, Tallahassee, FL, USA). For each pot, the final value recorded was the average of 5 measurements taken at different places.

On the 12^th^ day, coarse roots xylem and phloem, stem cortex and bark were also collected from control plants. All the organs were sampled in biological triplicate and snap-frozen in liquid nitrogen and stored at −80 °C before subsequent use.

### 4.2. Total RNA Isolation and cDNA Synthesis

Samples (250 mg) were ground into a fine powder using mortar and pestle in liquid nitrogen. Total RNA was extracted using a modified CTAB-buffer extraction protocol [44]. Total RNA was purified and treated with DNase I using the RNeasy plant mini kit (Qiagen, Leusden, The Netherlands) according to the manufacturer’s instructions. RNA purity and quantity were assessed by measuring the absorbance at 230, 260, and 280 nm using a Nanodrop ND1000 spectrophotometer (Thermo Scientific, Villebon-sur-Yvette, France). Total RNAs integrity was assessed using the RNA Nano 6000 Assay (Agilent Technologies, Diegem, Belgium) and a 2100 Bioanalyzer (Agilent technologies, Santa Clara, CA, USA) with parameters adapted to plant RNA profiles. All samples had sufficient purity and integrity scores. Precise RINs as well as 230/260 and 280/260 ratios are available in Table S5. Subsequently, one microgram of total RNA was retro-transcribed using ProtoScript II First Strand cDNA Synthesis Kit (NEB, Leiden, The Netherlands), following the manufacturer’s instructions.

### 4.3. Primer Design & qPCR Conditions

The sequences of genes used in this study were obtained from *Salix purpurea* genome annotation ver. 1.0 (Phytozome ver. 12.1.5; https://phytozome.jgi.doe.gov/pz/portal.html). All the sequences were blasted against *S. viminalis* EST (GenBank; https://www.ncbi.nlm.nih.gov/genbank/) and, when available, the top hit sequence was used for primer design. Primers were designed using Primer3Plus [45] with the following parameters: primer size 18–23 bases, amplicon length 60–200 bp, primer melting temperature 58–61 °C, CG content 40–60%. Primers were analyzed with OligoAnalyzer 3.1 (available online: https://eu.idtdna.com/calc/analyzer) to detect potential self- and hetero-dimers.

For each primer pair, standard curves were obtained using a 5-fold dilution series of cDNA template over seven dilution points, starting from a concentration of 12.5 ng/µL. The coefficient of determination (r^2^) and slope (S) values were obtained from the standard curves. All the coefficients of determination were above 0.99 (Table 2, Table S6). Primer efficiencies were calculated with qBase^PLUS^ (ver. 3.5; https://www.qbaseplus.com; Hellemans *et al.*, 2007 [46]). All efficiencies ranged from 83% to 115%.

For the qPCR analysis, 4 ng of cDNA were used as a template. The reactions were performed in a 384-wells plate prepared with a liquid handling robot (epMotion 5073, Eppendorf, Hamburg, Germany). The cDNA was amplified using Takyon low ROX SYBR qPCR MasterMix dTTP Blue Kit (Eurogentech, Liège, Belgium) on a Viia 7 Real-Time PCR System (Thermo Fisher, Waltham, MA, USA) in a final volume of 10 µL. No-template as well as non-RT controls ensured that the samples were free from contamination. The reactions were performed in technical triplicates for each biological independent replicate. The PCR conditions consisted of an initial denaturation at 95 °C for 3 min, followed by 40 cycles of denaturation at 95 °C for 10 sec and annealing/extension at 60 °C for 60 sec. A melting curve analysis was performed at the end of each experiment to check the specificity of the amplified products.

### 4.4. Analysis of Gene Expression Stability

#### 4.4.1. Descriptive Analyses

For all the analyses, data obtained from the Viia 7 Real-Time PCR Software were trimmed and exported into Excel datasheets. The descriptive analyses were performed using R (v. 3.5.1; https://www.r-project.org). The boxblots of the quantification cycle values were made with the median value of each technical replicate using the package ggplot2 (v. 3.2.0; https://cran.r-project.org/web/packages/ggplot2/). Stability over time was assessed by plotting the median value of each technical replicate as a function of the days. Linear regression and 95% confidence intervals were calculated by ggplot2.

#### 4.4.2. Determination of RG Expression Stability with geNormPLUS, BestKeeper, NormFinder, and GrayNorm

The geNorm^PLUS^ version included in qBase^PLUS^ was used, with the primer efficiencies previously calculated and all parameters set to default. As the original BestKeeper Excel spreadsheet (ver. 1.0; https://www.gene-quantification.de/bestkeeper.html) can only compute gene stability for a maximum of 10 candidate RGs, the R-based package ctrlGene (ver. 1.0.0; https://cran.r-project.org/web/packages/ctrlGene/) was used after verifying it yielded the same results as the Excel-based solution (cf. Results). NormFinder software (ver. 0.953; http://moma.dk/normfinder-software) is a Visual Basic Application based on Excel to rank RG expression stability. Input data was first transformed as required and a unique group identifier was set for each combination of organ, sampling day and condition. GrayNorm (ver. 1.1; https://github.com/gjbex/GrayNorm) is a python-based (ver. 2.7.x) algorithm able to compute the combination of genes that introduces the least uncertainty during gene expression normalization. It was used on Anaconda (ver. 1.9.2; https://www.anaconda.com/distribution/) and control groups were defined as the samples from day 0, with no treatment. When RG stability in more than one organ was computed, roots were also set as control group.

### 4.5. Consensus Ranking of Candidate RGs with RankAggreg

In order to generate a consensus from the data produced by geNorm^PLUS^, BestKeeper, NormFinder, and GrayNorm, we aggregated the obtained ranking lists by applying the RankAggreg (ver. 0.6.5) package of R software as done previously [16,43,47]. RankAggreg is a package which provides algorithms able to combine different ranking lists. Based on the size of our ranking lists, we used the Cross-Entropy Monte Carlo algorithm. The ranking list previously generated were used as input with the following parameters: Distance was calculated using the Spearman’s Footrule function, *rho* was set at 0.1, the seed at 75, and the “convIn” argument at 50.

### 4.6. Reference Genes Validation

The expression level of the stress-responsive genes studied in the various organs and conditions was analyzed using qBase^PLUS^ and normalized using the five most stable RGs as indicated in the “Results” section. The expression level of the stress-responsive genes, expressed as “Calibrated Normalized Relative Quantities” (CNRQs) as calculated by qBase^PLUS^, was exported to Excel datasheet. A one-way ANOVA (followed by a Tukey-Kramer post-hoc test) taking all the possible “day x condition” combination as factors was performed on the log_Efficiency_ transformed CNRQs using R and the agricolae package (https://cran.r-project.org/web/packages/agricolae).

## 5. Conclusions

The expression stability of 14 candidate RGs was assessed in *Salix viminalis* leaves and roots exposed for 12 days to five abiotic stress conditions. This assessment was done using four algorithms and the different rankings they produced were aggregated into a consensus list. Out of the 14 candidate RGs, *TIP41* and *CDC2* were globally the most stable genes across all conditions. Other RGs that should be used in combination with *TIP41* and *CDC2* were organ and condition dependent. Our results provide a panel of reference genes that can be used when performing a gene expression analysis in different organs and under various stresses in *S. viminalis*. These reference genes will be useful for unwinding the expression patterns and function of genes involved in stress tolerance. This report also emphasizes the MIQE recommendations. Amongst those, it clearly outlines the importance to use more than one reference gene, which unfortunately still occurs a lot. In addition, this report emphasizes the importance to validate widely used reference genes before performing an experiment with specific plants and stresses.

## Figures and Tables

**Table 1:** (**a**) Sampling time of the different organs of 8 months old willows exposed to different abiotic stress factors for 12 days. R: roots, L: leaves, O: other organs (roots stele, roots cortex, stem xylem and bark). (**b**) Evolution of the F_V_/F_M_ ratios during the time course of the experiment. Values given are the averaged F_V_/F_M_ ratios ± standard deviation of the measures made on three biological replicates. On day 7, only one plant exposed to drought stress still had its leaves. Dark green, F_V_/F_M_ ratio between 0.825 and 0.850; light green, 0.800–0.825; yellow, 0.775–0.800; orange, 0.750–0.775; red, F_V_/F_M_ ratio under 0.750. (**c**) Evolution of the soil relative water content (RWC) in the soil of control and drought-exposed plants. Plants exposed to salt, cold, and heat stress were watered at the same frequency as control plants.

(a)						
**Time Point (Day After Stress Exposure)**	**0**	**1**	**4**	**6**	**7**	**12**
Control	R+L	R+L	R+L		R+L	R+L+O
Salt		R+L	R+L			R+L
Cold		R+L	R+L		R+L	
Heat		R+L	R+L		R+L	
Drought		R+L	R+L	R+L	R+L	
						
	**Two months**					
Control	R+L					
Metals	R+L					
(b)						
**Time Point (Day After Stress Exposure)**	**0**	**1**	**4**	**6**	**7**	**12**
Control	0.821 ± 0.017	0.841 ± 0.010	0.812 ± 0.021	0.837 ± 0.001	0.803 ± 0.037	0.800 ± 0.027
Salt		0.848 ± 0.004	0.836 ± 0.003		0.832 ± 0.007	0.805 ± 0.015
Cold		0.770 ± 0.038	0.824 ± 0.012		0.831 ± 0.016	
Heat		0.806 ± 0.014	0.790 ± 0.013		0.759 ± 0.068	
Drought		0.832 ± 0.016	0.816 ± 0.038	0.806 ± 0.024	0.817	
						
	**Two months**					
Control	0.821 ± 0.017					
Metals	0.610 ± 0.060					
(c)						
**Time Point (Day After Stress Exposure)**		**0**	**1**	**4**	**6**	**7**
Control		33.14 ± 1.53	27.63 ± 4.81	49.37 ± 3.46	50.13 ± 2.39	58.87 ± 4.29
Drought			23.80 ± 1.42	3.80 ± 0.99	2.93 ± 0.52	2.43 ± 0.45

**Table 2:** List of candidate reference genes used in this study. Gene abbreviation, gene description, accession number, amplicon size, primer sequence, melting temperature (T_M_), primer efficiency, and coefficient of determination (r^2^) are given. The first three accession numbers, which correspond to *S. viminalis* EST, are from GeneBank; the other accession numbers are from Phytozome and correspond to *S. purpurea* sequences.

Abbreviation	Gene Description	Accession Number	Primer (F/R 5′-3′) Sequence	Ampli. Size	TM	Efficiency	r^2^
*CDC2*	Cyclin Dependent Kinase-putative	CB185210.1 S_VIM2.A04	AGAAGATCCGTTTGGAGCAG	124	83.1	97.67	0.998
			TTCTCACTGTGCACCACATC				
*CYP*	Cyclophilin-putative	CB185309.1 S_VIM3.B02	GGACCTGGTGTGCTATCCAT	166	84.0	94.85	0.998
			TTCCAGATCCAACCTTCTCG				
*eTIF5*	Eukaryotic translation initiation factor 5 A	CB185347.1 S_VIM3.E04	GTTTCGACCTCCAAAACTGG	124	81.2	97.36	0.997
			CATGGGGAACATCACAGTTG				
*α-TUB*	α-Tubulin subunit 2	SapurV1A.0598s0030.1	TGCCAAGTGACACCTCAATC	123	81.7	99.42	0.999
			CATCAATGACAGTGGGTTCG				
*β-TUB*	β-Tubulin	SapurV1A.1459s0040.1	GTGACTCGGCTCTCCAACTC	183	83.0	88.37	0.990
			TACCAGCACCAGATTGACCA				
*ACT*	Actin	SapurV1A.0231s0320.1	GATTGGATCTTGCTGGTCGT	150	83.3	87.09	0.996
			GCTCCTGCTCGTAGTCAAGG				
*ARI8*	E3 Ubiquitin protein ligase-putative	SapurV1A.0557s0250.1	TTACATGCACACCACCTTGC	93	81.7	95.75	0.992
			ATGCGTAAAAGCCACCTGTC				
*EF1b*	Elongation Factor 1-β	SapurV1A.1951s0030.1	AGTTTCTCGTCGGCAAATCC	88	78.6	94.84	0.997
			CCAGGTTTCTCCAAAACAGC				
*GAPDH*	Glyceraldehyde-3-posphate dehydrogenase	SapurV1A.0266s0210.1	TGTTGACTTCCGATGCTCTG	116	81.3	91.22	0.998
			GGCTGTATCCCCATTCATTG				
*OTUp*	OTU-like cysteine protease	SapurV1A.0615s0200.1	TCCAAGGTGGAAGGTGAAAG	80	80.5	103.90	0.994
			CCCATTGACAGCAACATCTG				
*PT1*	Unknown function, expressed in pollen tube	SapurV1A.0361s0260.1	CGCAAACAAAAACTGCAAGA	158	83.7	113.75	0.995
			ACTTCATCAGGCACCCAAAG				
*TIP41*	Type 2A phosphatase activator	SapurV1A.0019s0010.1	AACTGGCTGGAAACAAGAGG	131	82.5	97.28	0.994
			TACCACAATAAGGCGTCGTG				
*UCEE2*	Ubiquitin conjugating enzyme E2	SapurV1A.0237s0020.1	ATCATGGGTCCTCCTGATAGTC	109	81.8	83.86	0.998
			CCTTTGTCCTGAAAGCAACC				
*VHAC*	Vacuolar H+-ATPase subunit C	SapurV1A.0123s0450.1	TTGATGGTGTGCCAGTTGAC	148	81.4	90.18	0.991
			TCAGCAACACGAACCTTGAG				

**Table 3:** Potential reference genes (RGs) to normalize qPCR data in *Salix viminalis* roots exposed to various abiotic stresses. Plants were exposed to the stresses for up to two months. RGs expression stability is given by the “Average expression stability M” value as determined by geNorm^PLUS^. Lower stability value means greater stability. Stab.: gene stability value.

Rank	Control		Metals		Salt		Cold		Heat		Drought		Conditions Merged	
	Gene	Stab.	Gene	Stab.	Gene	Stab.	Gene	Stab.	Gene	Stab.	Gene	Stab.	Gene	Stab.
1	*CYP*	0.196	*CDC2*	0.232	*CDC2*	0.250	*CYP*	0.217	*ACT*	0.278	*CDC2*	0.247	*CDC2*	0.301
2	*eTIF5*	0.196	*TIP41*	0.239	*TIP41*	0.253	*CDC2*	0.222	*CDC2*	0.281	*ACT*	0.260	*TIP41*	0.307
3	*EF1b*	0.203	*α-TUB*	0.243	*CYP*	0.258	*eTIF5*	0.227	*TIP41*	0.283	*TIP41*	0.266	*ACT*	0.326
4	*CDC2*	0.223	*EF1b*	0.265	*ARI8*	0.273	*EF1b*	0.232	*eTIF5*	0.293	*ARI8*	0.291	*ARI8*	0.356
5	*ACT*	0.249	*ACT*	0.293	*EF1b*	0.298	*ACT*	0.248	*ARI8*	0.330	*CYP*	0.304	*EF1b*	0.377
6	*TIP41*	0.265	*ARI8*	0.316	*α-TUB*	0.310	*TIP41*	0.267	*VHAC*	0.351	*VHAC*	0.332	*UCEE2*	0.404
7	*β-TUB*	0.279	*CYP*	0.333	*ACT*	0.325	*α-TUB*	0.278	*EF1b*	0.372	*UCEE2*	0.353	*VHAC*	0.425
8	*α-TUB*	0.290	*OTUp*	0.365	*eTIF5*	0.338	*ARI8*	0.293	*UCEE2*	0.390	*EF1b*	0.386	*α-TUB*	0.454
9	*ARI8*	0.310	*UCEE2*	0.395	*VHAC*	0.355	*OTUp*	0.311	*PT1*	0.405	*PT1*	0.420	*CYP*	0.478
10	*VHAC*	0.331	*VHAC*	0.420	*UCEE2*	0.367	*UCEE2*	0.334	*α-TUB*	0.420	*α-TUB*	0.460	*PT1*	0.500
11	*UCEE2*	0.348	*eTIF5*	0.448	*OTUp*	0.380	*VHAC*	0.358	*OTUp*	0.449	*OUTp*	0.502	*OTUp*	0.526
12	*PT1*	0.360	*PT1*	0.473	*PT1*	0.399	*β-TUB*	0.380	*CYP*	0.483	*eTIF5*	0.545	*eTIF5*	0.549
13	*OTUp*	0.372	*β-TUB*	0.539	*β-TUB*	0.433	*PT1*	0.404	*β-TUB*	0.538	*GAPDH*	0.649	*GAPDH*	0.673
14	*GAPDH*	0.462	*GAPDH*	0.613	*GAPDH*	0.528	*GAPDH*	0.497	*GAPDH*	0.636	*β-TUB*	0.868	*β-TUB*	0.811

**Table 4:** Potential RGs to normalize qPCR data in *Salix viminalis* roots exposed to various abiotic stresses. Plants were exposed to the stresses for up to two months. Gene expression stability was calculated with BestKeeper. Two stability values are given: standard deviation SD [+/− C_q_] (the lower, the better) and by the coefficient of determination (r^2^) (the closer to 1, the better). Genes with SD [+/− Cq] over 1 should be discarded. Color scale: the bluer, the more stable.

Conditions	*α-TUB*	*ACT*	*ARI8*	*β-TUB*	*CDC2*	*CYP*	*EF1b*	*eTIF5*	*GAPDH*	*OTUp*	*PT1*	*TIP41*	*UCEE2*	*VHAC*
Control														
SD[+/− C_q_]	0.54	0.542	0.536	0.733	0.525	0.629	0.605	0.667	1.066	0.572	0.638	0.49	0.77	0.642
r^2^	0.816	0.865	0.873	0.823	0.965	0.969	0.885	0.938	0.405	0.832	0.909	0.94	0.927	0.905
*p*-value	0.001	0.001	0.001	0.001	0.001	0.001	0.001	0.001	0.026	0.001	0.001	0.001	0.001	0.001
Metals														
SD[+/− C_q_]	0.462	0.509	0.477	1.019	0.446	0.622	0.518	0.692	1.023	0.529	0.676	0.433	0.759	0.661
r^2^	0.786	0.751	0.865	0.436	0.877	0.896	0.884	0.635	0.352	0.698	0.781	0.899	0.873	0.77
*p*-value	0.001	0.001	0.001	0.007	0.001	0.001	0.001	0.001	0.02	0.001	0.001	0.001	0.001	0.001
Salt														
SD[+/− C_q_]	0.423	0.524	0.531	0.745	0.426	0.615	0.516	0.655	1.038	0.484	0.509	0.471	0.63	0.557
r^2^	0.782	0.768	0.852	0.59	0.925	0.921	0.857	0.843	0.321	0.724	0.779	0.89	0.888	0.852
*p*-value	0.001	0.001	0.001	0.001	0.001	0.001	0.001	0.001	0.014	0.001	0.001	0.001	0.001	0.001
Cold														
SD[+/− C_q_]	0.543	0.644	0.62	0.872	0.601	0.704	0.688	0.728	1.129	0.602	0.824	0.577	0.871	0.853
r^2^	0.857	0.904	0.892	0.839	0.934	0.964	0.907	0.933	0.387	0.866	0.889	0.907	0.909	0.893
*p*-value	0.001	0.001	0.001	0.001	0.001	0.001	0.001	0.001	0.003	0.001	0.001	0.001	0.001	0.001
Heat														
SD[+/− C_q_]	0.842	0.737	0.635	1.341	0.689	1.115	0.881	0.72	0.955	0.644	0.866	0.635	1.05	0.803
r^2^	0.892	0.915	0.869	0.805	0.97	0.891	0.931	0.923	0.034	0.648	0.954	0.898	0.957	0.906
*p*-value	0.001	0.001	0.001	0.001	0.001	0.001	0.001	0.001	0.422	0.001	0.001	0.001	0.001	0.001
Drought														
SD[+/− C_q_]	0.972	0.693	0.716	2.421	0.779	0.656	0.875	0.589	1.347	0.678	0.93	0.642	0.896	0.669
r^2^	0.777	0.9	0.895	0.654	0.941	0.891	0.918	0.36	0.145	0.551	0.931	0.924	0.899	0.87
*p*-value	0.001	0.001	0.001	0.001	0.001	0.001	0.001	0.007	0.108	0.001	0.001	0.001	0.001	0.001
Conditions merged														
SD[+/− C_q_]	0.821	0.748	0.68	1.746	0.672	0.912	0.812	0.694	1.364	0.605	0.87	0.584	0.866	0.757
r^2^	0.8	0.88	0.872	0.668	0.906	0.826	0.934	0.615	0.102	0.617	0.877	0.898	0.898	0.791
*p*-value	0.001	0.001	0.001	0.001	0.001	0.001	0.001	0.001	0.021	0.001	0.001	0.001	0.001	0.001

**Table 5:** Potential RGs to normalize qPCR data in *Salix viminalis* roots exposed to various abiotic stresses. Plants were exposed to the stresses for up to two months. Stability value of candidate RGs was determined by NormFinder. Genes are ranked from top to bottom in order of decreasing stability, lower values indicate higher stability. Stab.: gene stability value.

Rank	Control		Metals		Salt		Cold		Heat		Drought		Conditions Merged	
	Gene	Stab.	Gene	Stab.	Gene	Stab.	Gene	Stab.	Gene	Stab.	Gene	Stab.	Gene	Stab.
1	*CDC2*	0.102	*CYP*	0.150	*CDC2*	0.131	*CYP*	0.113	*CDC2*	0.124	*CDC2*	0.140	*CDC2*	0.200
2	*CYP*	0.106	*CDC2*	0.154	*TIP41*	0.162	*CDC2*	0.147	*eTIF5*	0.194	*TIP41*	0.193	*EF1b*	0.202
3	*TIP41*	0.154	*TIP41*	0.163	*ARI8*	0.172	*eTIF5*	0.153	*VHAC*	0.204	*CYP*	0.197	*TIP41*	0.214
4	*eTIF5*	0.166	*ARI8*	0.180	*CYP*	0.179	*ACT*	0.159	*ACT*	0.221	*ARI8*	0.208	*ARI8*	0.220
5	*VHAC*	0.169	*EF1b*	0.183	*VHAC*	0.192	*TIP41*	0.179	*TIP41*	0.224	*ACT*	0.226	*ACT*	0.233
6	*ARI8*	0.191	*ACT*	0.237	*EF1b*	0.196	*EF1b*	0.186	*UCEE2*	0.242	*VHAC*	0.229	*UCEE2*	0.247
7	*ACT*	0.199	*UCEE2*	0.264	*UCEE2*	0.202	*ARI8*	0.196	*ARI8*	0.257	*UCEE2*	0.232	*VHAC*	0.312
8	*UCEE2*	0.207	*α-TUB*	0.267	*ACT*	0.220	*OTUp*	0.206	*PT1*	0.268	*EF1b*	0.234	*CYP*	0.318
9	*EF1b*	0.208	*VHAC*	0.269	*α-TUB*	0.227	*UCEE2*	0.220	*EF1b*	0.275	*PT1*	0.318	*α-TUB*	0.348
10	*OTUp*	0.222	*OTUp*	0.286	*OTUp*	0.252	*VHAC*	0.233	*α-TUB*	0.280	*α-TUB*	0.420	*eTIF5*	0.353
11	*PT1*	0.232	*eTIF5*	0.305	*eTIF5*	0.253	*α-TUB*	0.234	*CYP*	0.403	*OTUp*	0.454	*PT1*	0.354
12	*α-TUB*	0.246	*PT1*	0.334	*PT1*	0.264	*PT1*	0.300	*OTUp*	0.403	*eTIF5*	0.497	*OTUp*	0.385
13	*β-TUB*	0.257	*β-TUB*	0.442	*β-TUB*	0.289	*β-TUB*	0.303	*β-TUB*	0.544	*GAPDH*	0.830	*β-TUB*	0.765
14	*GAPDH*	0.504	*GAPDH*	0.549	*GAPDH*	0.544	*GAPDH*	0.513	*GAPDH*	0.652	*β-TUB*	1.148	*GAPDH*	0.823

**Table 6:** Potential RGs to normalize qPCR data in *Salix viminalis* roots exposed to various abiotic stresses. Plants were exposed to the stresses for up to two months. Gene expression stability was calculated with GrayNorm. Genes are ranked according to their internal coefficient of variation (CV_inter_). Only single gene combinations are shown.

Rank	Control		Metals		Salt		Cold		Heat		Drought		Conditions Merged	
	Gene	CV_inter_	Gene	CV_inter_	Gene	CV_inter_	Gene	CV_inter_	Gene	CV_inter_	Gene	CV_inter_	Gene	CV_inter_
1	*ACT*	0.479	*ACT*	0.427	*ACT*	0.472	*OTUp*	0.483	*OTUp*	0.563	*VHAC*	0.490	*OTUp*	0.563
2	*TIP41*	0.497	*ARI8*	0.451	*TIP41*	0.485	*TIP41*	0.495	*ARI8*	0.617	*CYP*	0.514	*GAPDH*	0.610
3	*ARI8*	0.506	*TIP41*	0.465	*ARI8*	0.493	*ACT*	0.495	*GAPDH*	0.610	*ACT*	0.516	*ARI8*	0.617
4	*OTUp*	0.556	*VHAC*	0.498	*EF1b*	0.501	*ARI8*	0.497	*TIP41*	0.640	*TIP41*	0.537	*VHAC*	0.639
5	*VHAC*	0.578	*CYP*	0.505	*VHAC*	0.505	*CDC2*	0.535	*CDC2*	0.723	*OTUp*	0.558	*TIP41*	0.640
6	*EF1b*	0.580	*EF1b*	0.521	*β-TUB*	0.530	*EF1b*	0.538	*eTIF5*	0.768	*eTIF5*	0.601	*CDC2*	0.723
7	*CDC2*	0.581	*CDC2*	0.555	*GAPDH*	0.538	*CYP*	0.547	*ACT*	0.736	*ARI8*	0.601	*ACT*	0.736
8	*CYP*	0.586	*OTUp*	0.565	*CDC2*	0.550	*α-TUB*	0.557	*VHAC*	0.639	*UCEE2*	0.611	*eTIF5*	0.768
9	*α-TUB*	0.620	*α-TUB*	0.577	*α-TUB*	0.551	*eTIF5*	0.596	*α-TUB*	0.932	*GAPDH*	0.616	*UCEE2*	0.854
10	*eTIF5*	0.653	*PT1*	0.592	*OTUp*	0.554	*VHAC*	0.617	*UCEE2*	0.854	*CDC2*	0.650	*PT1*	0.921
11	*β-TUB*	0.660	*UCEE2*	0.648	*PT1*	0.558	*GAPDH*	0.661	*PT1*	0.921	*PT1*	0.807	*α-TUB*	0.932
12	*PT1*	0.702	*eTIF5*	0.673	*CYP*	0.591	*UCEE2*	0.675	*EF1b*	1.045	*EF1b*	0.824	*EF1b*	1.045
13	*GAPDH*	0.719	*GAPDH*	0.771	*UCEE2*	0.670	*β-TUB*	0.688	*CYP*	1.458	*α-TUB*	0.869	*CYP*	1.458
14	*UCEE2*	0.766	*β-TUB*	0.778	*eTIF5*	0.687	*PT1*	0.715	*β-TUB*	2.147	*β-TUB*	1.795	*β-TUB*	2.147

**Table 7:** Consensus ranking of the 14 candidate RGs to normalize qPCR data in *Salix viminalis* roots exposed to various abiotic stresses for up to two months. Plants were exposed to the stresses for up to two months. This consensus ranking list was generated by RankAggreg. Genes are listed from top to bottom in order of decreasing expression stability.

Rank	Control	Metals	Salt	Cold	Heat	Drought	Conditions Merged
1	*CYP*	*TIP41*	*CDC2*	*CYP*	*CDC2*	*CDC2*	*CDC2*
2	*CDC2*	*CDC2*	*TIP41*	*CDC2*	*TIP41*	*TIP41*	*TIP41*
3	*TIP41*	*ARI8*	*ARI8*	*TIP41*	*ARI8*	*CYP*	*ARI8*
4	*ARI8*	*EF1b*	*EF1b*	*ACT*	*eTIF5*	*ACT*	*ACT*
5	*ACT*	*ACT*	*CYP*	*eTIF5*	*ACT*	*VHAC*	*EF1b*
6	*VHAC*	*CYP*	*VHAC*	*EF1b*	*VHAC*	*ARI8*	*UCEE2*
7	*EF1b*	*α-TUB*	*ACT*	*OTUp*	*UCEE2*	*UCEE2*	*VHAC*
8	*eTIF5*	*OTUp*	*α-TUB*	*ARI8*	*PT1*	*EF1b*	*CYP*
9	*α-TUB*	*VHAC*	*UCEE2*	*α-TUB*	*EF1b*	*PT1*	*α-TUB*
10	*OTUp*	*PT1*	*OTUp*	*UCEE2*	*α-TUB*	*α-TUB*	*PT1*
11	*PT1*	*eTIF5*	*PT1*	*VHAC*	*OTUp*	*OTUp*	*OTUp*
12	*β-TUB*	*UCEE2*	*eTIF5*	*PT1*	*CYP*	*eTIF5*	*eTIF5*
13	*UCEE2*	*β-TUB*	*β-TUB*	*β-TUB*	*β-TUB*	*GAPDH*	*GAPDH*
14	*GAPDH*	*GAPDH*	*GAPDH*	*GAPDH*	*GAPDH*	*β-TUB*	*β-TUB*

**Table 8:** Optimal combinations of reference genes to use for appropriate data normalization in *S. viminalis* roots under abiotic stress conditions as computed by GrayNorm.

Conditions		CV_inter_
Control		
1	*ACT*	0.479
2	*TIP41* + *ACT*	0.490
3	*ARI8* + *ACT*	0.493
Metals		
1	*ACT*	0.427
2	*ARI8* + *ACT*	0.441
3	*ARI8* + *ACT* + *VHAC*	0.447
Salt		
1	*GAPDH* + *ACT*+ *β-Tub*	0.435
2	*TIP41* + *GAPDH* + *β-Tub*	0.441
3	*TIP41* + *GAPDH* + *ACT*+ *β-Tub*	0.443
Cold		
1	*TIP41* + *OTUp* + *ARI8* + *GAPDH* + *ACT*	0.474
2	*TIP41* + *ARI8* + *GAPDH* + *ACT*	0.475
3	*TIP41* + *OTUp* + *ARI8* + *GAPDH*	0.476
Heat		
1	*TIP41* + *GAPDH*	0.439
2	*GAPDH* + *ACT*	0.441
3	*CDC2* + *GAPDH*	0.456
Drought		
1	*GAPDH* + *ACT*+ *VHAC*	0.472
2	*GAPDH* + *ACT*	0.474
3	*eTIF5* + *ACT*+ *VHAC*	0.475
Conditions merged		
1	*GAPDH* + *VHAC*	0.454
2	*TIP41* + *GAPDH*	0.457
3	*TIP41* + *GAPDH* + *VHAC*	0.465

## Supplementary Materials

The following are available online at https://www.mdpi.com/1422-0067/20/17/4210/s1.
