# Supplementary material for: Selection of Appropriate Reference Genes for Gene Expression Analysis under Abiotic Stresses in Salix viminalis
Source: Int J Mol Sci. 2019 Aug 28;20(17):4210. doi: 10.3390/ijms20174210 (PMC6747362; doi:10.3390/ijms20174210)
Supplement: Supplementary file 1 [file ijms-20-04210-s001.zip › supplementary caption.docx]

Supplementary Figure 1. Melting curves of the final qPCR products of the candidate RGs and stress-responsive genes.
Supplementary Figure 2. Quantification cycle values of the 14 candidate RGs as a function of time and stress conditions in the root.
Supplementary Figure 3. Quantification cycle values of the 14 candidate RGs as a function of time and stress conditions in the leaves.
Supplementary Figure 4. Normalized relative expression of the three stress-responsive genes in the leaves.
Supplementary Table 1. Precise chlorophyll fluorescence and relative water content value at each time points.
Supplementary Table 2. Consensus ranking list of the 14 candidate RGs to be used in the leaves.
Supplementary Table 3. Consensus ranking list of the 12 candidate RGs to be used in the roots and leaves mixed together.
Supplementary Table 4. Consensus ranking list of the 12 candidate RGs to be used in the roots, leaves, coarse roots stele and cortex, stem xylem and bark mixed together.
Supplementary Table 5. RNA quality, purity and integrity.
Supplementary Table 6. Information on the stress-responsive genes. Abbreviations are the same as in Table 2.
