# Supplementary material for: Selection of Appropriate Reference Genes for Gene Expression Analysis under Abiotic Stresses in Salix viminalis
Source: Int J Mol Sci. 2019 Aug 28;20(17):4210. doi: 10.3390/ijms20174210 (PMC6747362; doi:10.3390/ijms20174210)
Supplement: Supplementary file 1 [file ijms-20-04210-s001.zip › Supp_Table.2-RankAggreg_Leaves_Consensus.docx]

| Rank | Control | Metals | Salt | Cold | Heat | Drought | Conditions merged |
| --- | --- | --- | --- | --- | --- | --- | --- |
| 1 | *TIP41* | *EF1b* | *CDC2* | *EF1b* | *CDC2* | *TIP41* | *ARI8* |
| 2 | *ARI8* | *ARI8* | *TIP41* | *TIP41* | *TIP41* | *EF1b* | *TIP41* |
| 3 | *EF1b* | *TIP41* | *ARI8* | *CDC2* | *ARI8* | *UCEE2* | *UCEE2* |
| 4 | *UCEE2* | *ACT* | *EF1b* | *eTIF5* | *OTUp* | *ACT* | *OTUp* |
| 5 | *eTIF5* | *CDC2* | *eTIF5* | *ARI8* | *eTIF5* | *OTUp* | *CDC2* |
| 6 | *CDC2* | *UCEE2* | *ACT* | *UCEE2* | *EF1b* | *a-TUB* | *EF1b* |
| 7 | *a-TUB* | *OTUp* | *VHAC* | *OTUp* | *UCEE2* | *ARI8* | *eTIF5* |
| 8 | *OTUp* | *VHAC* | *PT1* | *a-TUB* | *ACT* | *VHAC* | *VHAC* |
| 9 | *GAPDH* | *PT1* | *UCEE2* | *VHAC* | *a-TUB* | *CDC2* | *ACT* |
| 10 | *PT1* | *eTIF5* | *GAPDH* | *ACT* | *PT1* | *eTIF5* | *PT1* |
| 11 | *ACT* | *a-TUB* | *a-TUB* | *PT1* | *VHAC* | *PT1* | *a-TUB* |
| 12 | *VHAC* | *GAPDH* | *OTUp* | *GAPDH* | *CYP* | *CYP* | *CYP* |
| 13 | *CYP* | *CYP* | *CYP* | *CYP* | *GAPDH* | *b-TUB* | *b-TUB* |
| 14 | *b-TUB* | *b-TUB* | *b-TUB* | *b-TUB* | *b-TUB* | *GAPDH* | *GAPDH* |
