# Supplementary material for: Selection of Appropriate Reference Genes for Gene Expression Analysis under Abiotic Stresses in Salix viminalis
Source: Int J Mol Sci. 2019 Aug 28;20(17):4210. doi: 10.3390/ijms20174210 (PMC6747362; doi:10.3390/ijms20174210)
Supplement: Supplementary file 1 [file ijms-20-04210-s001.zip › Supp_Table.4-RankAggreg_all_Organs_Consensus.docx]

| Rank | Organs |
| --- | --- |
| 1 | *eTIF5* |
| 2 | *TIP41* |
| 3 | *CDC2* |
| 4 | *EF1b* |
| 5 | *ARI8* |
| 6 | *VHAC* |
| 7 | *UCEE2* |
| 8 | *OTUp* |
| 9 | *ACT* |
| 10 | *CYP* |
| 11 | *PT1* |
| 12 | *α-TUB* |
