# Supplementary material for: Selection of Appropriate Reference Genes for Gene Expression Analysis under Abiotic Stresses in Salix viminalis
Source: Int J Mol Sci. 2019 Aug 28;20(17):4210. doi: 10.3390/ijms20174210 (PMC6747362; doi:10.3390/ijms20174210)
Supplement: Supplementary file 1 [file ijms-20-04210-s001.zip › Supp_Table.6-Stress_Genes_Info_Table.docx]

| **Abbreviation** | **Gene description** | **Accession number** | **Primer (F/R 5'-3') sequence** | **Ampli. size** | **TM** | **Efficiency** | **r2** |
| --- | --- | --- | --- | --- | --- | --- | --- |
| *ADC* | Arginine decarboxylase | SapurV1A.0091s0150 | TGGCTATGAACTGCTTGTGC | 105 | 82.2 | 96.631 | 0.993 |
|  |  |  | AGAGTTTCCTCGCAATCAGC |  |  |  |  |
| *CAT* | Catalase | SapurV1A.0016s0660 | CGATGGGTTGATGCCTTATC | 123 | 83.4 | 101.74 | 0.996 |
|  |  |  | TTTCACGCTGAGACGAGATG |  |  |  |  |
| *HSP17* | Heat Shock Protein of 17.6 kDa | SapurV1A.0393s0170 | GAAATCAGGGGACATCAAGG | 82 | 81.2 | 103.72 | 0.995 |
|  |  |  | TTTATCCTCTCCACGCTTCC |  |  |  |  |
