# Supplementary material for: Selection of Appropriate Reference Genes for Gene Expression Analysis under Abiotic Stresses in Salix viminalis
Source: Int J Mol Sci. 2019 Aug 28;20(17):4210. doi: 10.3390/ijms20174210 (PMC6747362; doi:10.3390/ijms20174210)
Supplement: Supplementary file 1 [file ijms-20-04210-s001.zip › Table.2-Genes_Info_Table.docx]

| **Abbreviation** | **Gene description** | **Accession number** | **Primer (F/R 5'-3') sequence** | **Ampli. size** | **TM** | **Efficiency** | **r^2^** |
| --- | --- | --- | --- | --- | --- | --- | --- |
| *CDC2* | Cyclin Dependent Kinase - putative | CB185210.1 S_VIM2.A04 | AGAAGATCCGTTTGGAGCAG | 124 | 83.1 | 97.67 | 0.998 |
|  |  |  | TTCTCACTGTGCACCACATC |  |  |  |  |
| *CYP* | Cyclophilin - putative | CB185309.1 S_VIM3.B02 | GGACCTGGTGTGCTATCCAT | 166 | 84.0 | 94.85 | 0.998 |
|  |  |  | TTCCAGATCCAACCTTCTCG |  |  |  |  |
| *eTIF5* | Eukaryotic translation initiation factor 5 A | CB185347.1 S_VIM3.E04 | GTTTCGACCTCCAAAACTGG | 124 | 81.2 | 97.36 | 0.997 |
|  |  |  | CATGGGGAACATCACAGTTG |  |  |  |  |
| *α-TUB* | α-Tubulin subunit 2 | SapurV1A.0598s0030.1 | TGCCAAGTGACACCTCAATC | 123 | 81.7 | 99.42 | 0.999 |
|  |  |  | CATCAATGACAGTGGGTTCG |  |  |  |  |
| *β-TUB* | β-Tubulin | SapurV1A.1459s0040.1 | GTGACTCGGCTCTCCAACTC | 183 | 83.0 | 88.37 | 0.990 |
|  |  |  | TACCAGCACCAGATTGACCA |  |  |  |  |
| *ACT* | Actin | SapurV1A.0231s0320.1 | GATTGGATCTTGCTGGTCGT | 150 | 83.3 | 87.09 | 0.996 |
|  |  |  | GCTCCTGCTCGTAGTCAAGG |  |  |  |  |
| *ARI8* | E3 Ubiquitin protein ligase - putative | SapurV1A.0557s0250.1 | TTACATGCACACCACCTTGC | 93 | 81.7 | 95.75 | 0.992 |
|  |  |  | ATGCGTAAAAGCCACCTGTC |  |  |  |  |
| *EF1b* | Elongation Factor 1-β | SapurV1A.1951s0030.1 | AGTTTCTCGTCGGCAAATCC | 88 | 78.6 | 94.84 | 0.997 |
|  |  |  | CCAGGTTTCTCCAAAACAGC |  |  |  |  |
| *GAPDH* | Glyceraldehyde-3-posphate dehydrogenase | SapurV1A.0266s0210.1 | TGTTGACTTCCGATGCTCTG | 116 | 81.3 | 91.22 | 0.998 |
|  |  |  | GGCTGTATCCCCATTCATTG |  |  |  |  |
| *OTUp* | OTU-like cysteine protease | SapurV1A.0615s0200.1 | TCCAAGGTGGAAGGTGAAAG | 80 | 80.5 | 103.90 | 0.994 |
|  |  |  | CCCATTGACAGCAACATCTG |  |  |  |  |
| *PT1* | Unknown function, expressed in pollen tube | SapurV1A.0361s0260.1 | CGCAAACAAAAACTGCAAGA | 158 | 83.7 | 113.75 | 0.995 |
|  |  |  | ACTTCATCAGGCACCCAAAG |  |  |  |  |
| *TIP41* | Type 2A phosphatase activator | SapurV1A.0019s0010.1 | AACTGGCTGGAAACAAGAGG | 131 | 82.5 | 97.28 | 0.994 |
|  |  |  | TACCACAATAAGGCGTCGTG |  |  |  |  |
| *UCEE2* | Ubiquitin conjugating enzyme E2 | SapurV1A.0237s0020.1 | ATCATGGGTCCTCCTGATAGTC | 109 | 81.8 | 83.86 | 0.998 |
|  |  |  | CCTTTGTCCTGAAAGCAACC |  |  |  |  |
| *VHAC* | Vacuolar H+-ATPase subunit C | SapurV1A.0123s0450.1 | TTGATGGTGTGCCAGTTGAC | 148 | 81.4 | 90.18 | 0.991 |
|  |  |  | TCAGCAACACGAACCTTGAG |  |  |  |  |
