# Supplementary material for: Selection of Appropriate Reference Genes for Gene Expression Analysis under Abiotic Stresses in Salix viminalis
Source: Int J Mol Sci. 2019 Aug 28;20(17):4210. doi: 10.3390/ijms20174210 (PMC6747362; doi:10.3390/ijms20174210)
Supplement: Supplementary file 1 [file ijms-20-04210-s001.zip › Table.4-BK_Root_Results.docx]

| **Conditions** | ***α-TUB*** | ***ACT*** | ***ARI8*** | ***β-TUB*** | ***CDC2*** | ***CYP*** | ***EF1b*** | ***eTIF5*** | ***GAPDH*** | ***OTUp*** | ***PT1*** | ***TIP41*** | ***UCEE2*** | ***VHAC*** |
| --- | --- | --- | --- | --- | --- | --- | --- | --- | --- | --- | --- | --- | --- | --- |
| Control | | | | | | | | | | | | | | |
| SD[+/- C_q_] | 0.54 | 0.542 | 0.536 | 0.733 | 0.525 | 0.629 | 0.605 | 0.667 | 1.066 | 0.572 | 0.638 | 0.49 | 0.77 | 0.642 |
| r^2^ | 0.816 | 0.865 | 0.873 | 0.823 | 0.965 | 0.969 | 0.885 | 0.938 | 0.405 | 0.832 | 0.909 | 0.94 | 0.927 | 0.905 |
| p-value | 0.001 | 0.001 | 0.001 | 0.001 | 0.001 | 0.001 | 0.001 | 0.001 | 0.026 | 0.001 | 0.001 | 0.001 | 0.001 | 0.001 |
| Metals | | | | | | | | | | | | | | |
| SD[+/- C_q_] | 0.462 | 0.509 | 0.477 | 1.019 | 0.446 | 0.622 | 0.518 | 0.692 | 1.023 | 0.529 | 0.676 | 0.433 | 0.759 | 0.661 |
| r^2^ | 0.786 | 0.751 | 0.865 | 0.436 | 0.877 | 0.896 | 0.884 | 0.635 | 0.352 | 0.698 | 0.781 | 0.899 | 0.873 | 0.77 |
| p-value | 0.001 | 0.001 | 0.001 | 0.007 | 0.001 | 0.001 | 0.001 | 0.001 | 0.02 | 0.001 | 0.001 | 0.001 | 0.001 | 0.001 |
| Salt | | | | | | | | | | | | | | |
| SD[+/- C_q_] | 0.423 | 0.524 | 0.531 | 0.745 | 0.426 | 0.615 | 0.516 | 0.655 | 1.038 | 0.484 | 0.509 | 0.471 | 0.63 | 0.557 |
| r^2^ | 0.782 | 0.768 | 0.852 | 0.59 | 0.925 | 0.921 | 0.857 | 0.843 | 0.321 | 0.724 | 0.779 | 0.89 | 0.888 | 0.852 |
| p-value | 0.001 | 0.001 | 0.001 | 0.001 | 0.001 | 0.001 | 0.001 | 0.001 | 0.014 | 0.001 | 0.001 | 0.001 | 0.001 | 0.001 |
| Cold | | | | | | | | | | | | | | |
| SD[+/- C_q_] | 0.543 | 0.644 | 0.62 | 0.872 | 0.601 | 0.704 | 0.688 | 0.728 | 1.129 | 0.602 | 0.824 | 0.577 | 0.871 | 0.853 |
| r^2^ | 0.857 | 0.904 | 0.892 | 0.839 | 0.934 | 0.964 | 0.907 | 0.933 | 0.387 | 0.866 | 0.889 | 0.907 | 0.909 | 0.893 |
| p-value | 0.001 | 0.001 | 0.001 | 0.001 | 0.001 | 0.001 | 0.001 | 0.001 | 0.003 | 0.001 | 0.001 | 0.001 | 0.001 | 0.001 |
| Heat | | | | | | | | | | | | | | |
| SD[+/- C_q_] | 0.842 | 0.737 | 0.635 | 1.341 | 0.689 | 1.115 | 0.881 | 0.72 | 0.955 | 0.644 | 0.866 | 0.635 | 1.05 | 0.803 |
| r^2^ | 0.892 | 0.915 | 0.869 | 0.805 | 0.97 | 0.891 | 0.931 | 0.923 | 0.034 | 0.648 | 0.954 | 0.898 | 0.957 | 0.906 |
| p-value | 0.001 | 0.001 | 0.001 | 0.001 | 0.001 | 0.001 | 0.001 | 0.001 | 0.422 | 0.001 | 0.001 | 0.001 | 0.001 | 0.001 |
| Drought | | | | | | | | | | | | | | |
| SD[+/- C_q_] | 0.972 | 0.693 | 0.716 | 2.421 | 0.779 | 0.656 | 0.875 | 0.589 | 1.347 | 0.678 | 0.93 | 0.642 | 0.896 | 0.669 |
| r^2^ | 0.777 | 0.9 | 0.895 | 0.654 | 0.941 | 0.891 | 0.918 | 0.36 | 0.145 | 0.551 | 0.931 | 0.924 | 0.899 | 0.87 |
| p-value | 0.001 | 0.001 | 0.001 | 0.001 | 0.001 | 0.001 | 0.001 | 0.007 | 0.108 | 0.001 | 0.001 | 0.001 | 0.001 | 0.001 |
| Conditions merged | | | | | | | | | | | | | | |
| SD[+/- C_q_] | 0.821 | 0.748 | 0.68 | 1.746 | 0.672 | 0.912 | 0.812 | 0.694 | 1.364 | 0.605 | 0.87 | 0.584 | 0.866 | 0.757 |
| r^2^ | 0.8 | 0.88 | 0.872 | 0.668 | 0.906 | 0.826 | 0.934 | 0.615 | 0.102 | 0.617 | 0.877 | 0.898 | 0.898 | 0.791 |
| p-value | 0.001 | 0.001 | 0.001 | 0.001 | 0.001 | 0.001 | 0.001 | 0.001 | 0.021 | 0.001 | 0.001 | 0.001 | 0.001 | 0.001 |
