# Supplementary figures and images for: Selection of Appropriate Reference Genes for Gene Expression Analysis under Abiotic Stresses in Salix viminalis
Source: Int J Mol Sci. 2019 Aug 28;20(17):4210. doi: 10.3390/ijms20174210 (PMC6747362; doi:10.3390/ijms20174210)

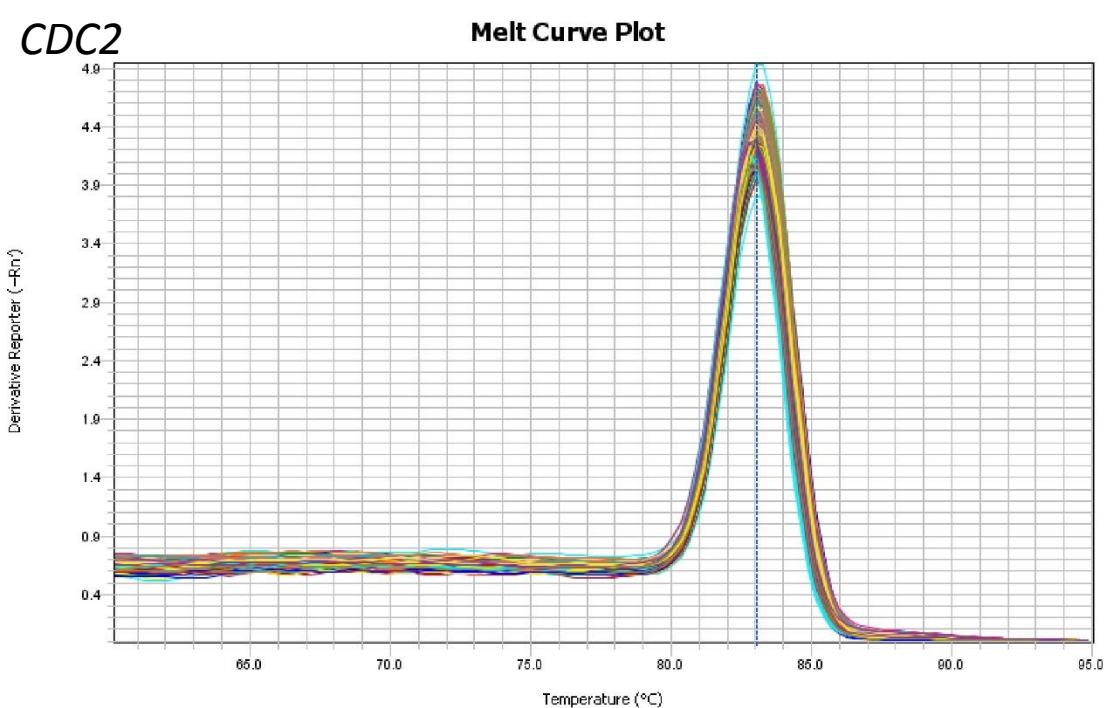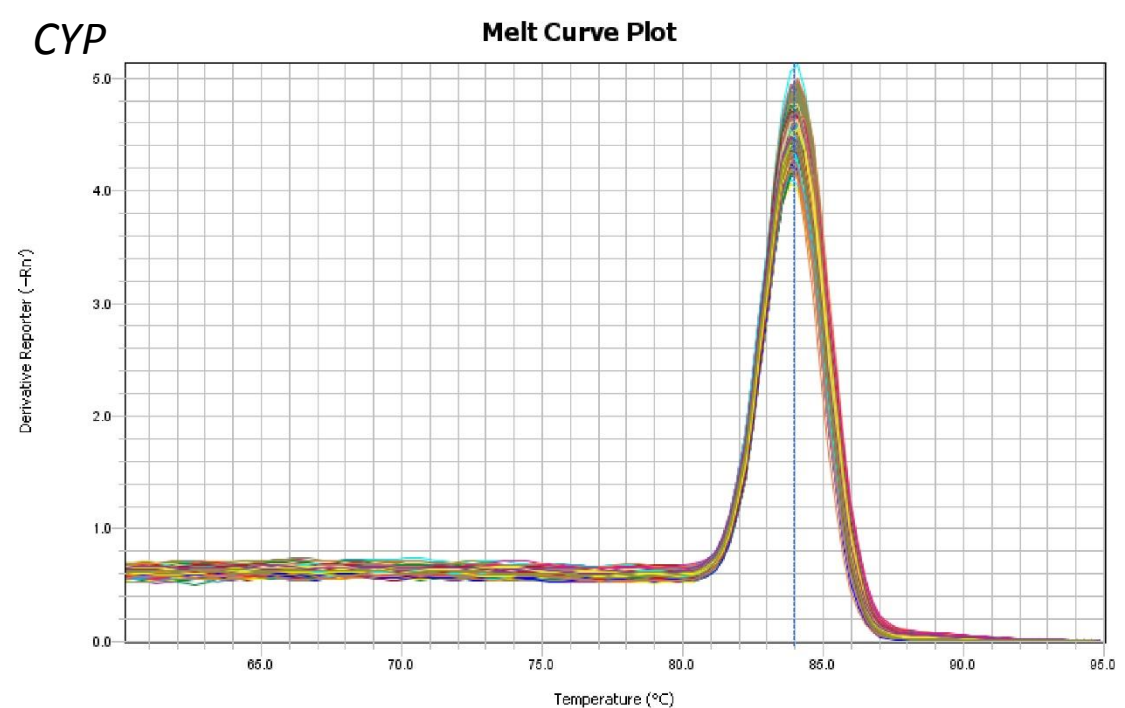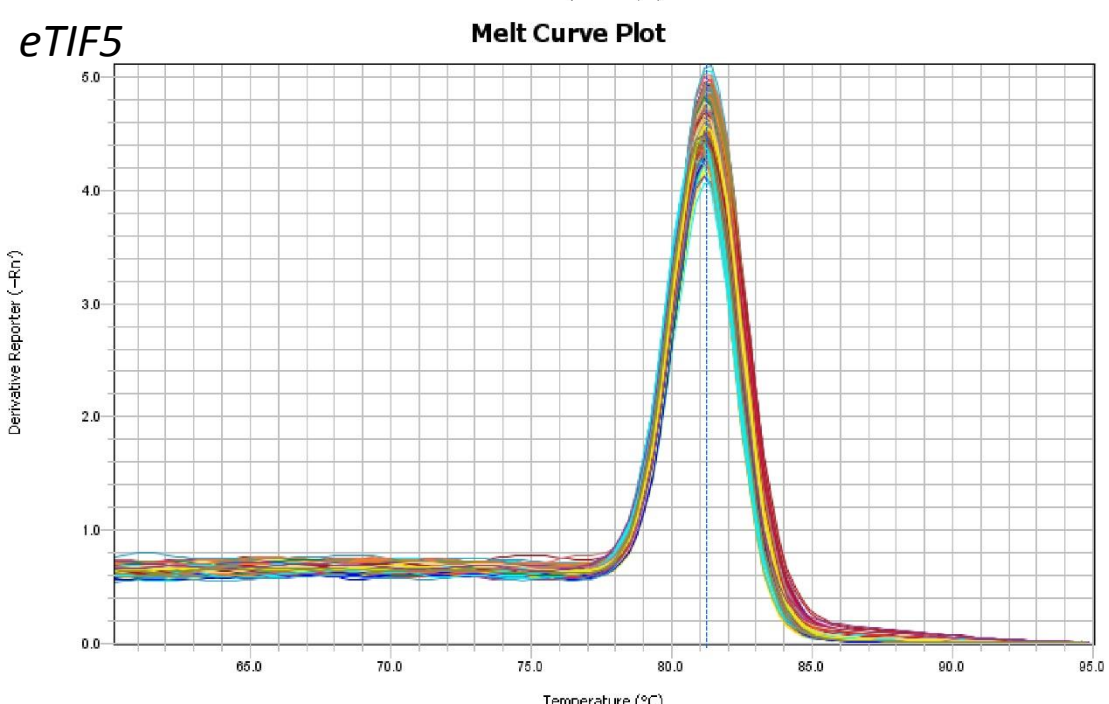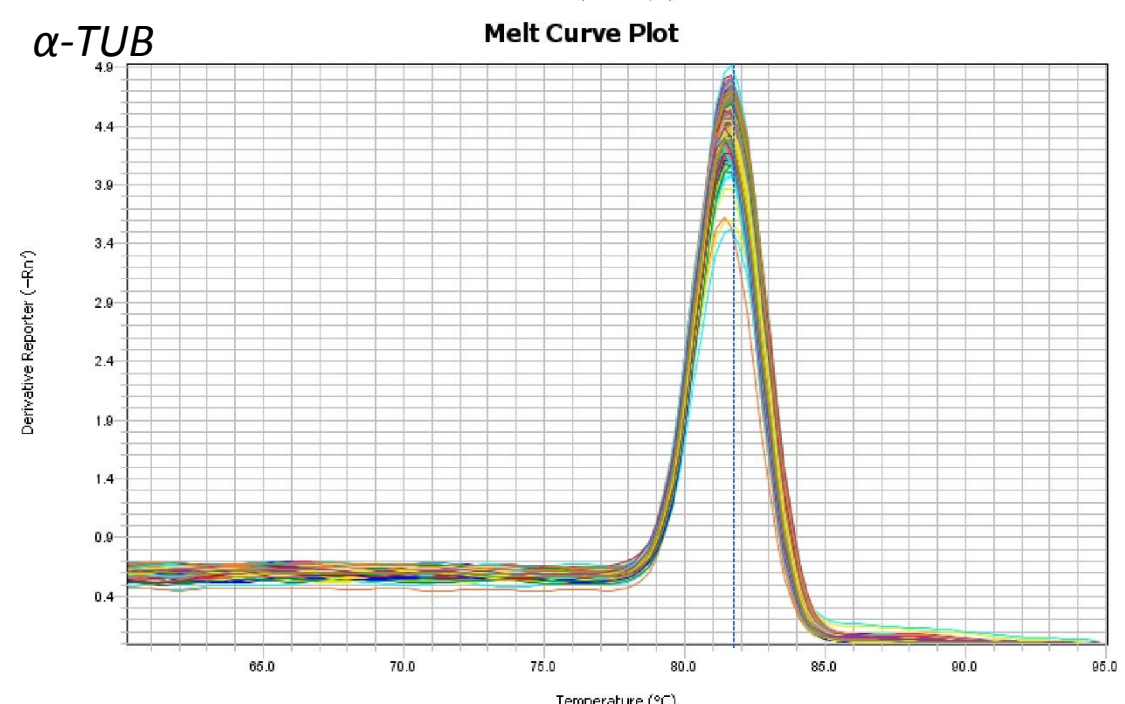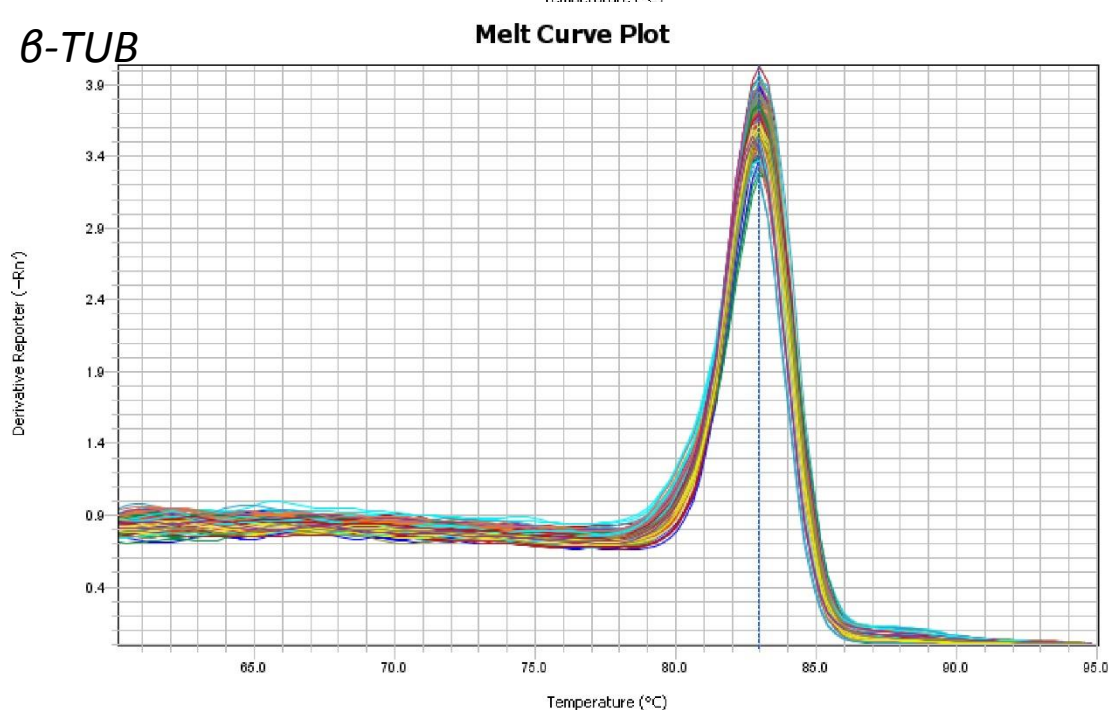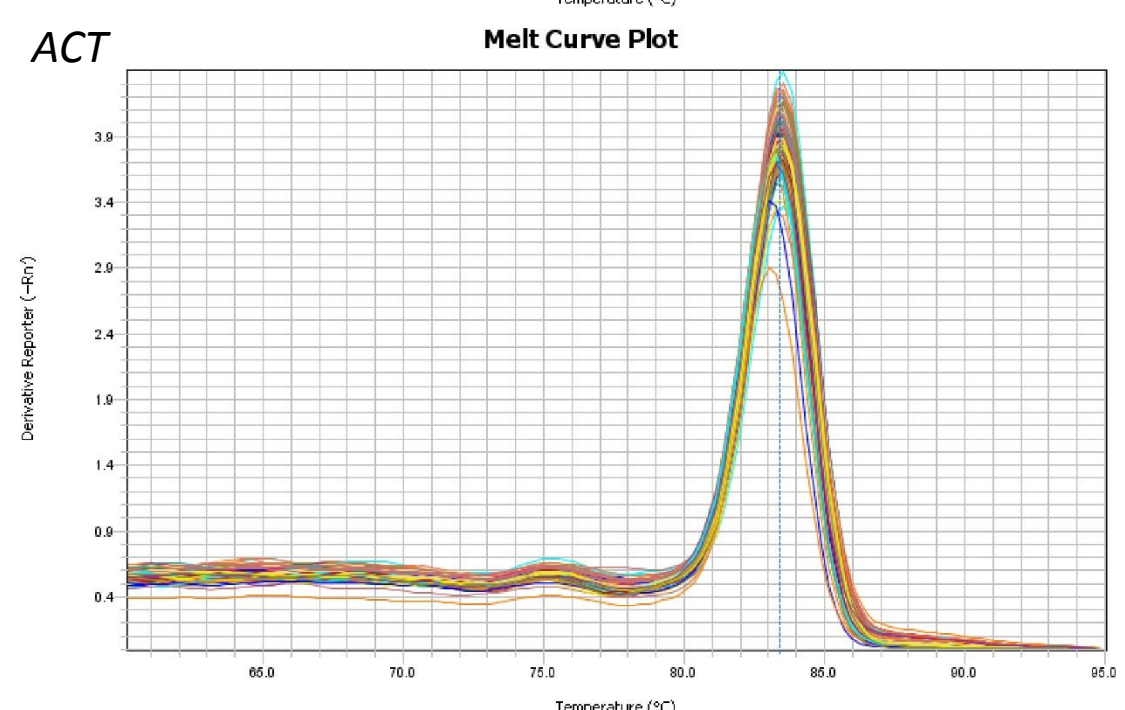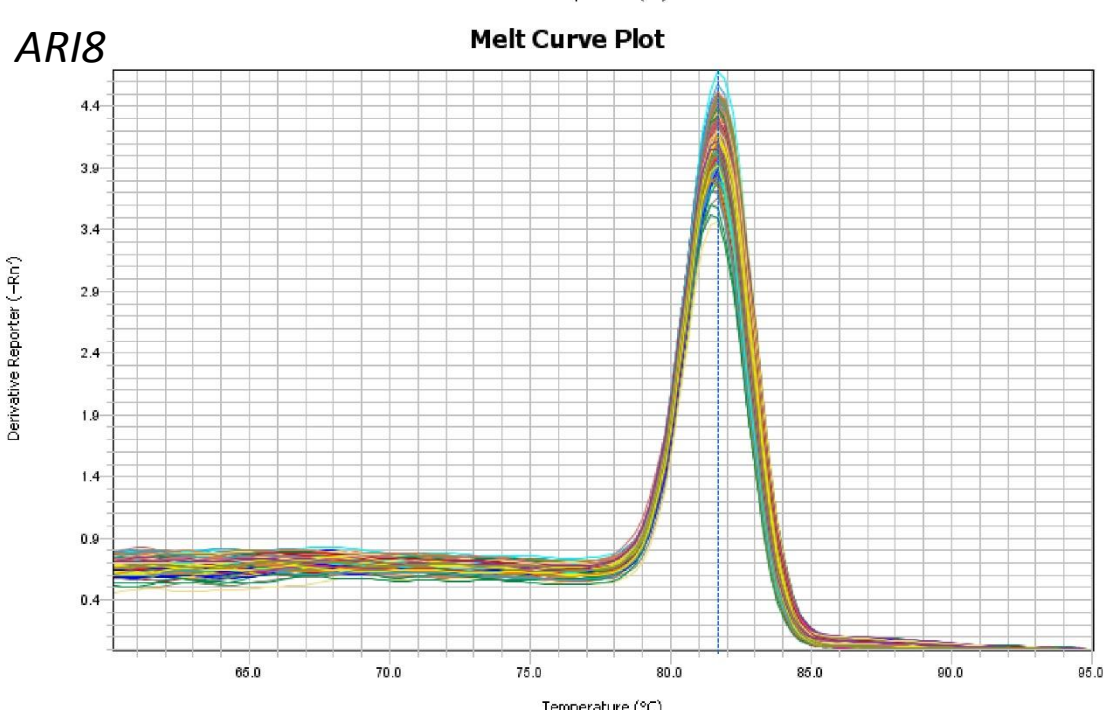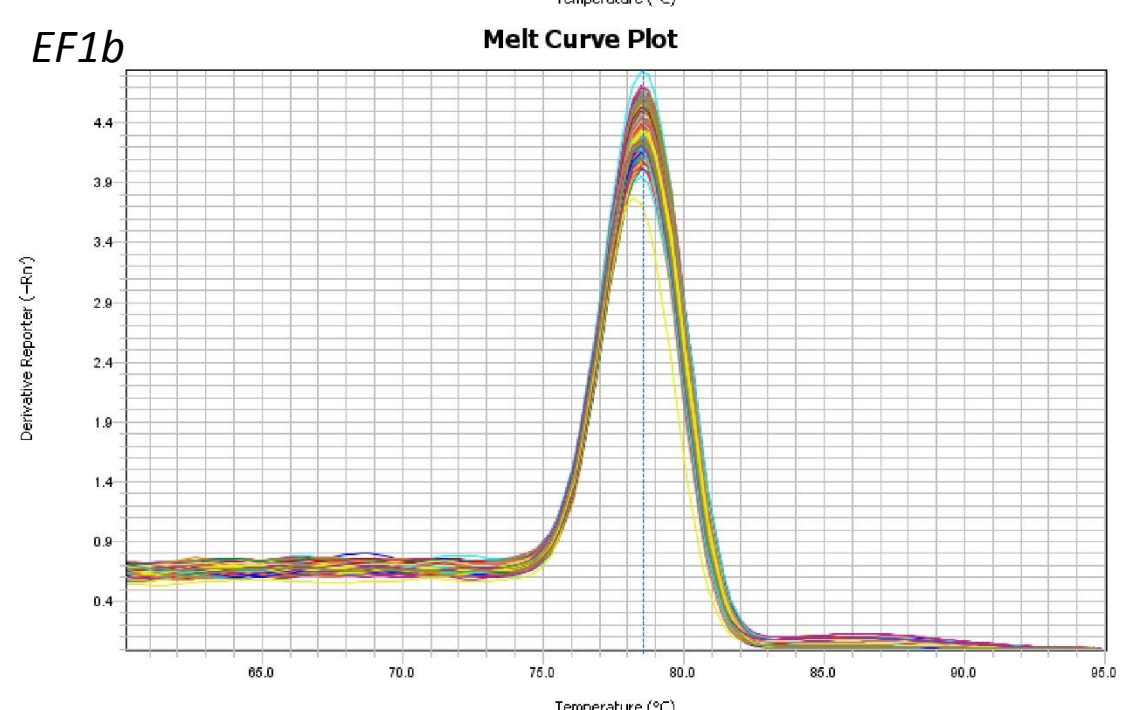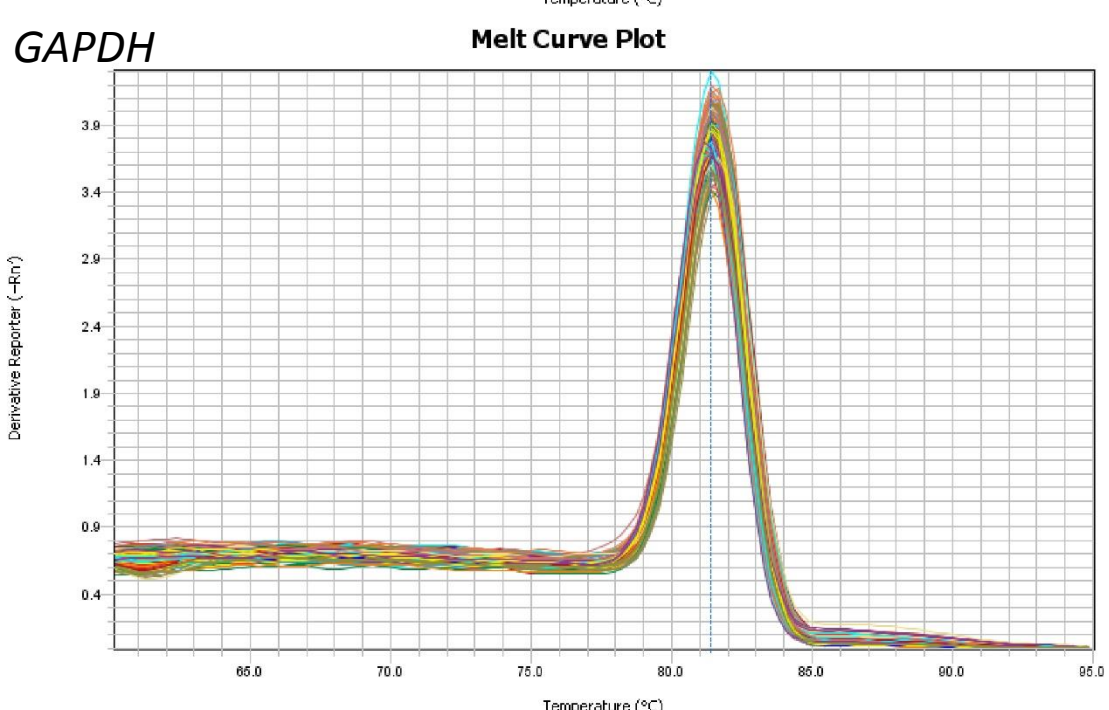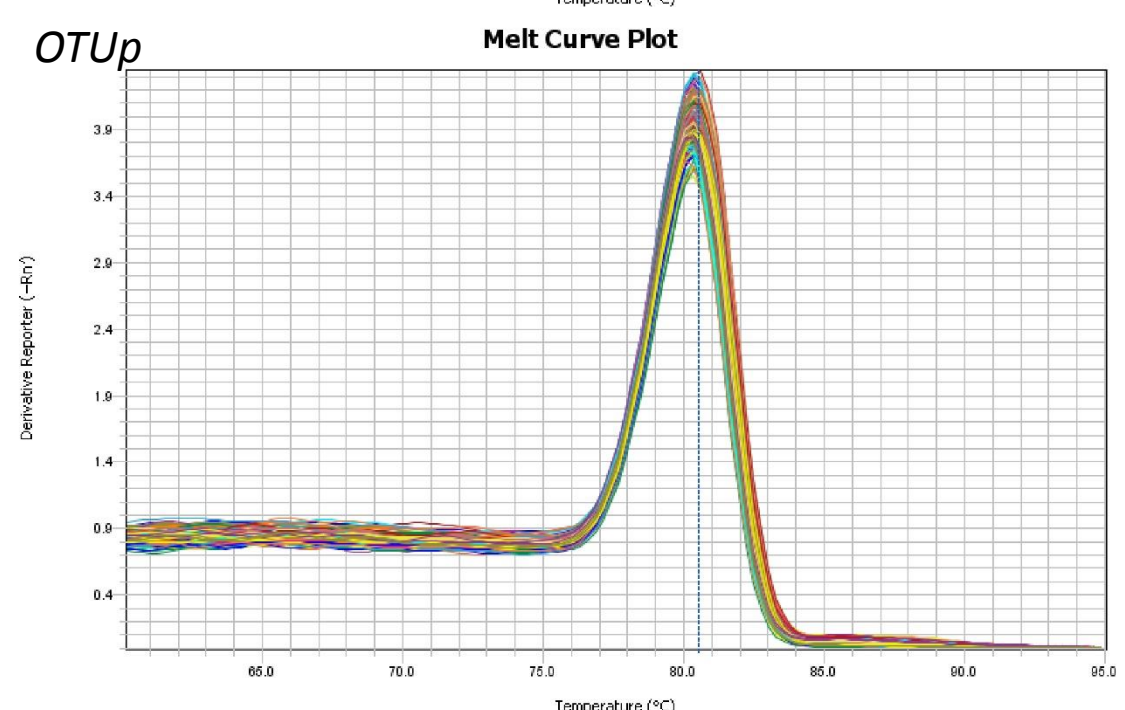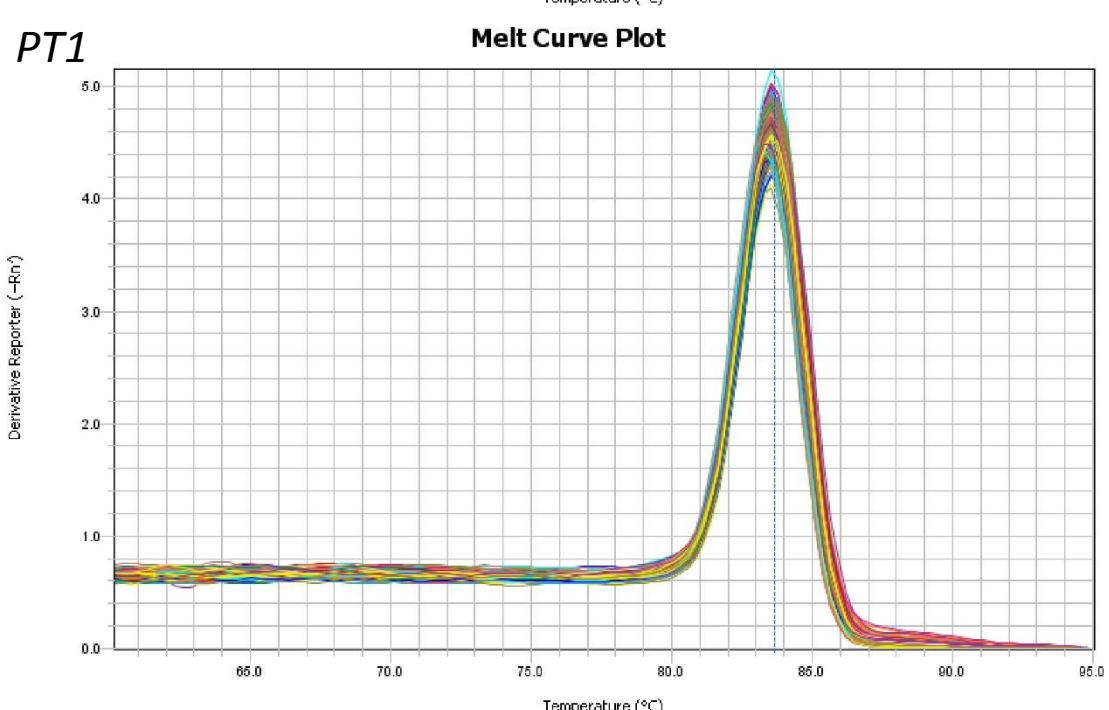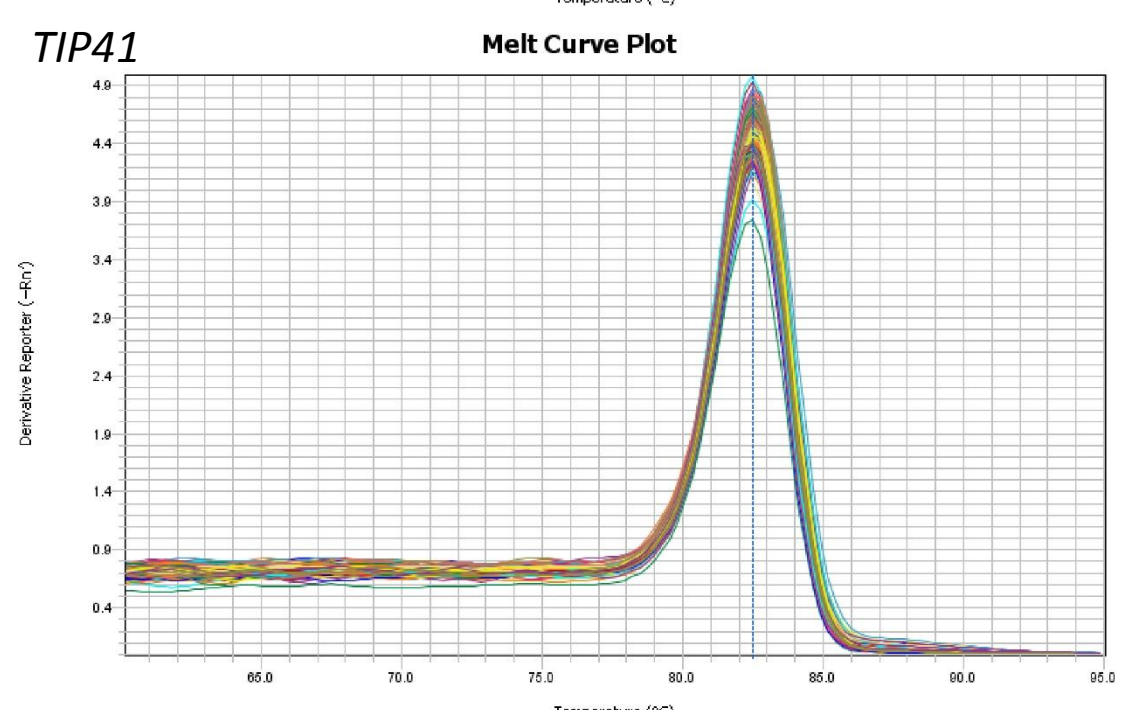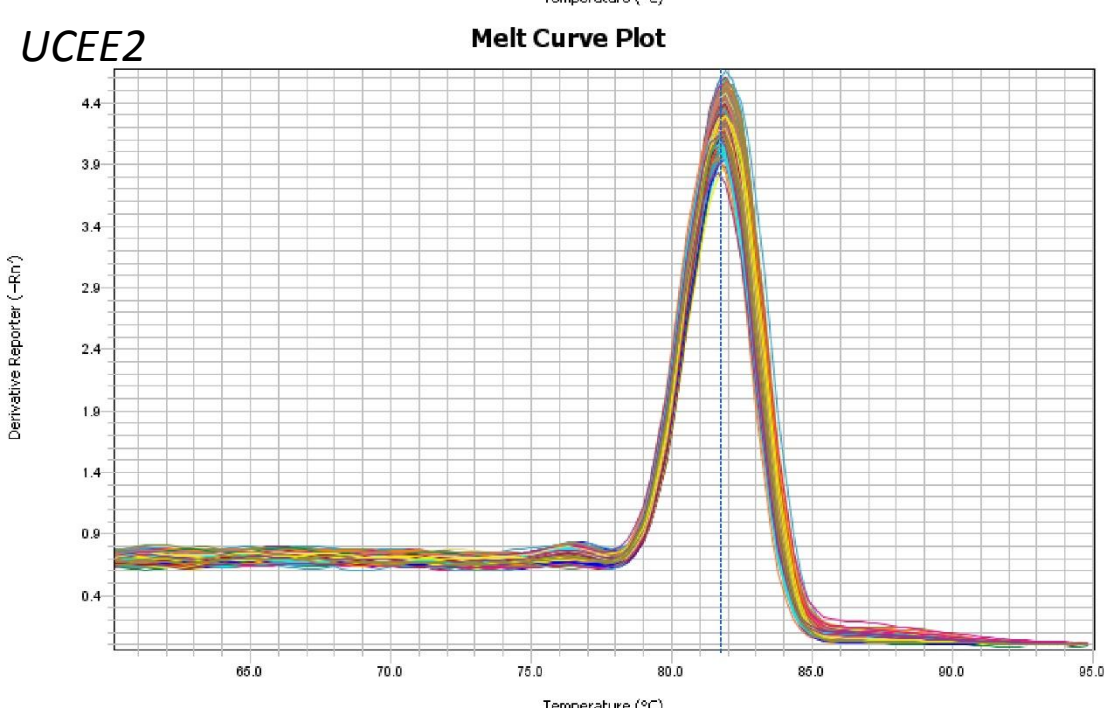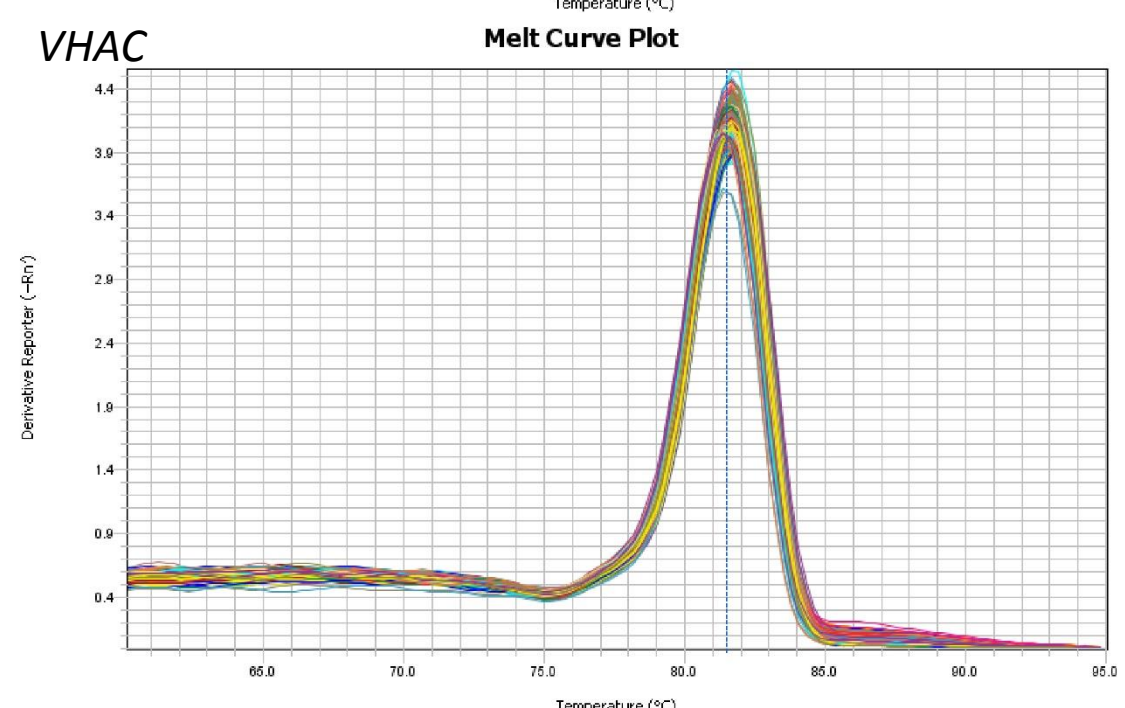

Supplement: Supplementary file 1 [file ijms-20-04210-s001.zip › Supp_Figure.1-Melting_curves.pdf]

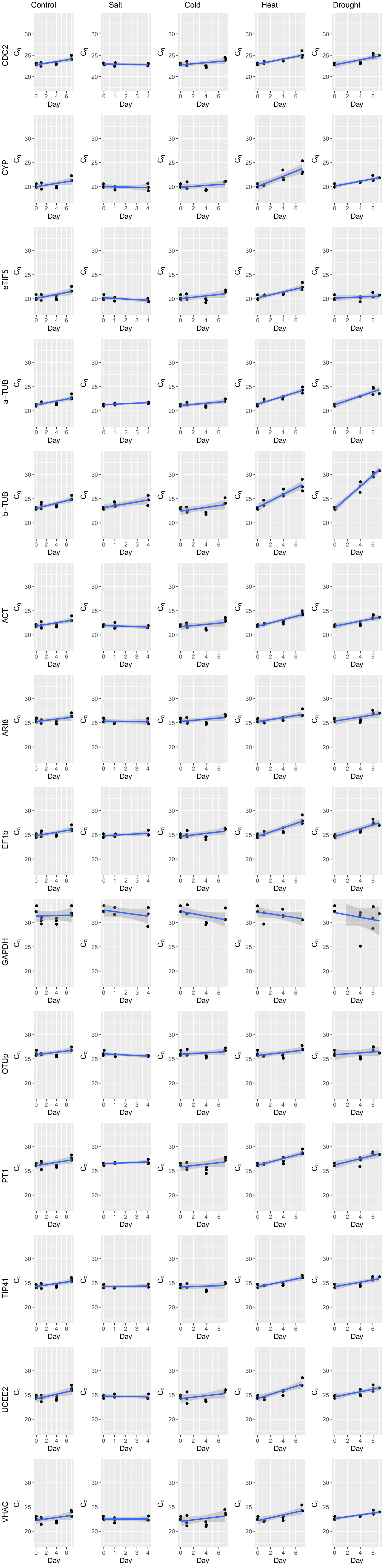

Supplement: Supplementary file 1 [file ijms-20-04210-s001.zip › Supp_Figure.2-Expression_level_over_time_roots.pdf]

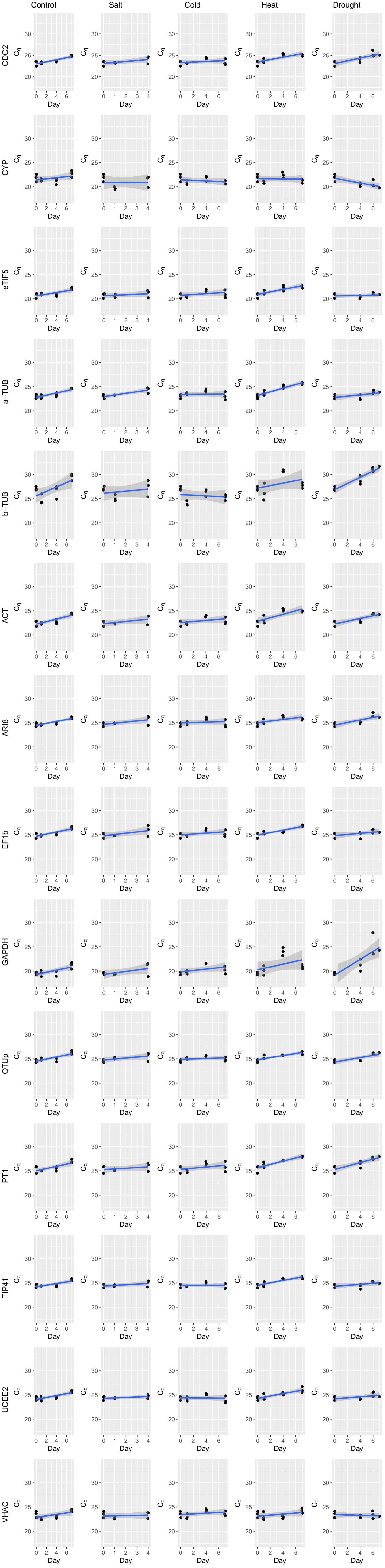

Supplement: Supplementary file 1 [file ijms-20-04210-s001.zip › Supp_Figure.3-Expression_level_over_time_leaves.pdf]

ADC

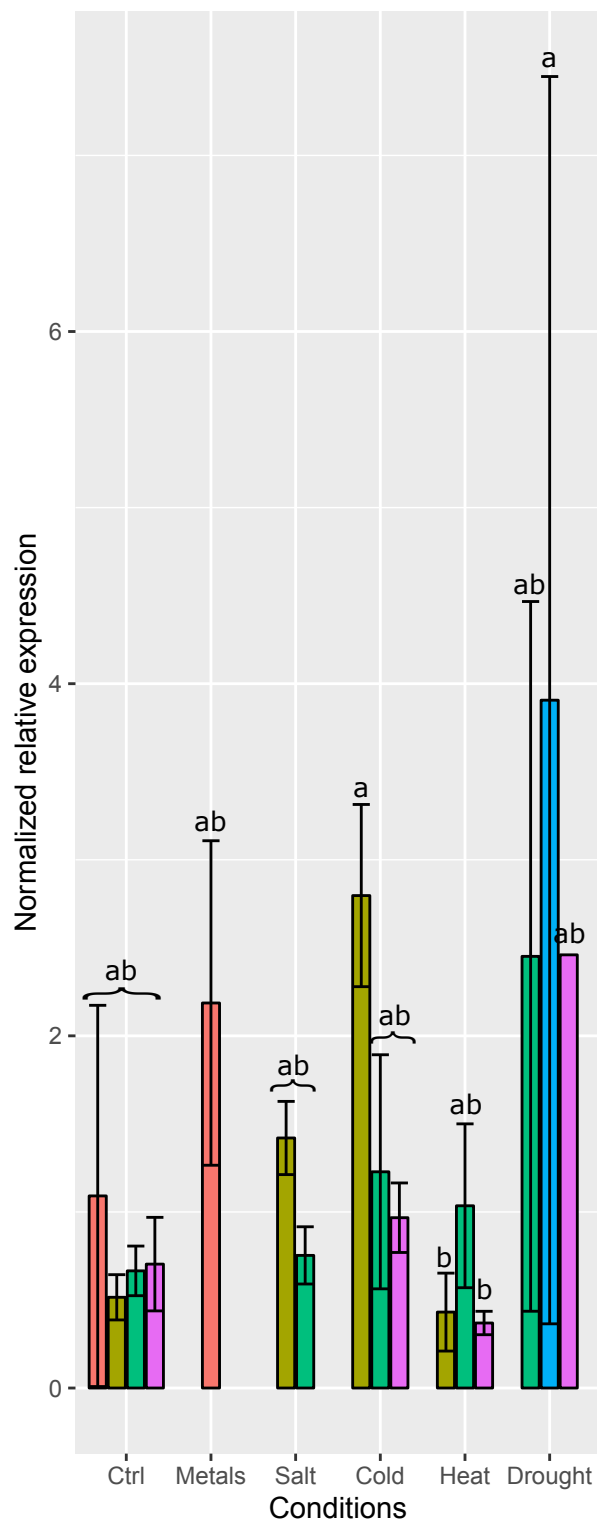

CAT

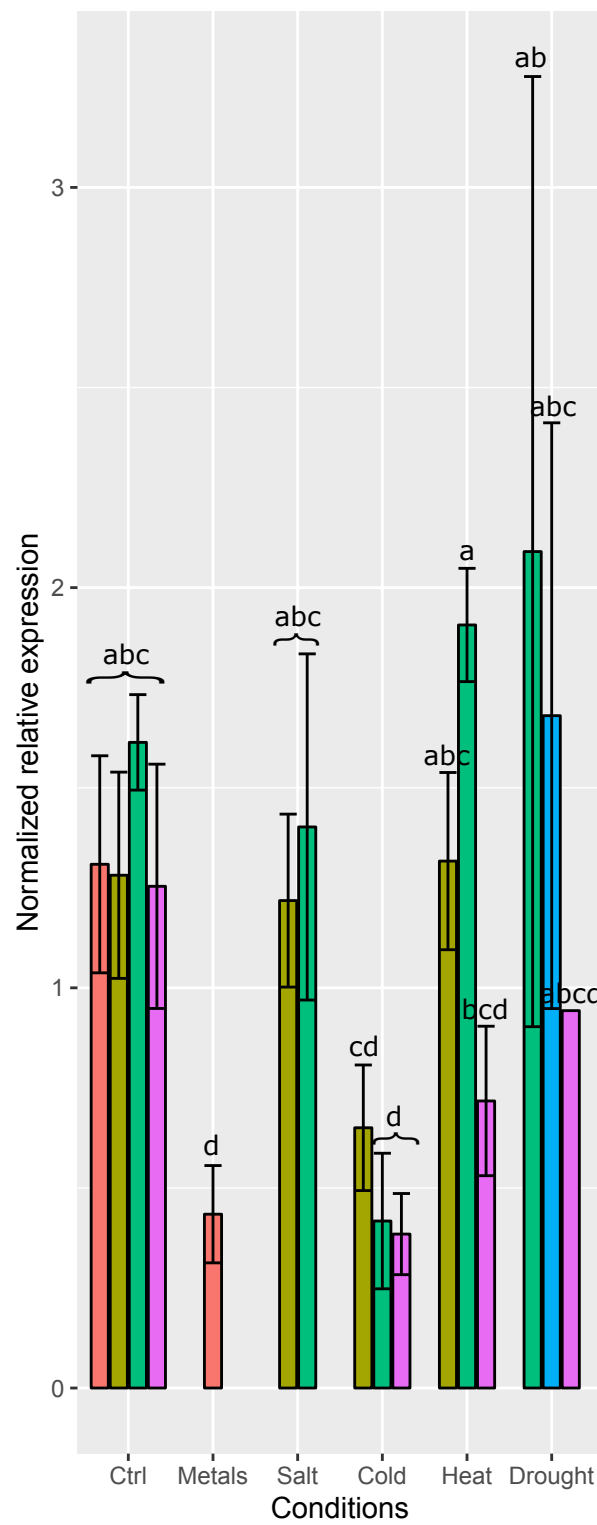

HSP17

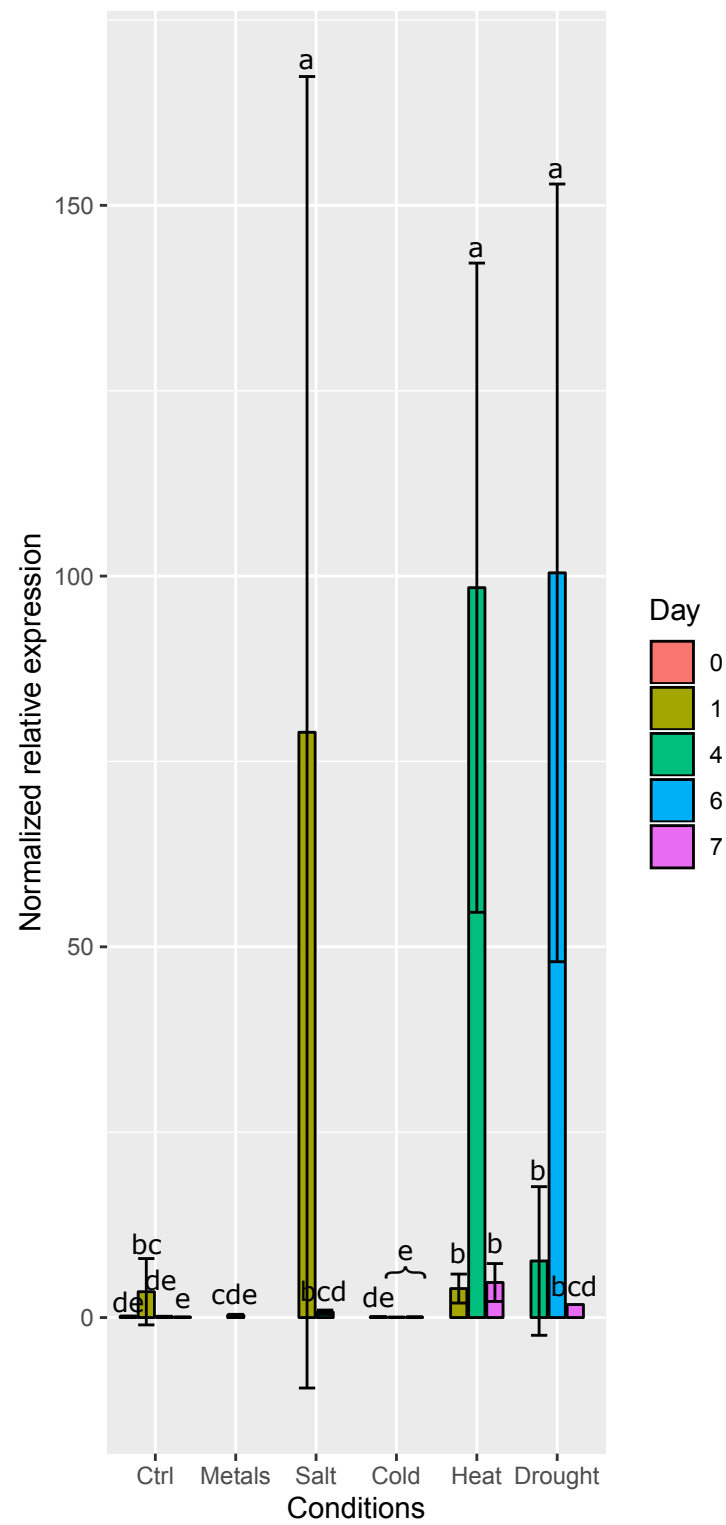

Supplement: Supplementary file 1 [file ijms-20-04210-s001.zip › Supp_Figure.4-Stress_Responsive_Leaves.pdf]
